# SPOP downregulation promotes bladder cancer progression based on cancer cell-macrophage crosstalk via STAT3/CCL2/IL-6 axis and is regulated by VEZF1

**DOI:** 10.7150/thno.101575

**Published:** 2024-10-07

**Authors:** Meiqian Li, Yangyan Cui, Qi Qi, Jiakuan Liu, Jiaxuan Li, Guifang Huang, Jiale Yang, Jingya Sun, Zhihui Ma, Shengjie Liang, Dianzheng Zhang, Jun Jiang, Rujian Zhu, Qiuli Liu, Ruimin Huang, Jun Yan

**Affiliations:** 1 Department of Urology, Shanghai Pudong Hospital, Fudan University Pudong Medical Center; Laboratory Animal Center, Fudan University, Shanghai 200032, China.; 2 MOE Key Laboratory of Model Animals for Disease Study, Model Animal Research Center of Nanjing University, Nanjing 210061, China.; 3 Laboratory Animal Center, Fudan University, Shanghai 200032, China.; 4 Shanghai Institute of Infectious Disease and Biosecurity, School of Public Health, Fudan University, Shanghai 200032, China.; 5 Center for Drug Safety Evaluation and Research, Shanghai Institute of Materia Medica, Chinese Academy of Sciences, Shanghai 201203, China.; 6 University of Chinese Academy of Sciences, Beijing 100049, China.; 7 Department of Bio-Medical Sciences, Philadelphia College of Osteopathic Medicine, Philadelphia, PA 19131, USA.; 8 Department of Urology, Daping Hospital, Army Medical University, Chongqing 400042, China.

**Keywords:** SPOP, bladder cancer, tumor-associated macrophage, STAT3/CCL2/IL-6 axis, VEZF1

## Abstract

**Background:** Cancer cells are intimately intertwined with tumor microenvironment (TME), fostering a symbiotic relationship propelling cancer progression. However, the interaction between cancer cells and tumor-associated macrophages (TAMs) in urothelial bladder cancer (UBC) remains poorly understood.

**Methods:** UBC cell lines (5637, T24 and SW780), along with a monocytic cell line (U937) capable of differentiating into macrophage, were used in a co-culture system for cell proliferation and stemness by MTT, sphere formation assays. VEZF1/SPOP/STAT3/CCL2/ IL-6 axis was determined by luciferase reporter, ChIP, RNA-seq, co-IP, *in vitro* ubiquitination, RT-qPCR array and ELISA analyses.

**Results:** We observed the frequent downregulation of SPOP, an E3 ubiquitin ligase, was positively associated with tumor progression and TAM infiltration in UBC patients and T24 xenografts. Cancer cell-TAM crosstalk promoting tumor aggressiveness was demonstrated dependent on SPOP deficiency: 1) In UBC cells, STAT3 was identified as a novel substrate of SPOP, and SPOP deficiency increased STAT3 protein stability, elevated chemokine CCL2 secretion, which induced chemotaxis and M2 polarization of macrophage; 2) In co-cultured macrophages, IL-6 secretion enhanced UBC cell proliferation and stemness. Additionally, transcription factor VEZF1 could directly activate SPOP transcription, and its overexpression suppressed the above effects in UBC cells.

**Conclusions:** A pivotal role of SPOP in maintaining UBC stemness and remodeling immunosuppressive TME was revealed. Both the intrinsic signaling (dysregulated VEZF1/SPOP/STAT3 axis) and the extrinsic cues from TME (CCL2-IL-6 axis based on macrophages) promoted UBC progression. Targeting this crosstalk may offer a promising therapeutic strategy for UBC patients with SPOP deficiency.

## Introduction

Urothelial bladder cancer (UBC) stands as one of the most aggressive malignancies, characterized by its high rates of recurrence and mortality. In 2024, an estimated 82,290 new cases of UBC and 16,710 UBC-related deaths are expected in the United States 1. More critically, there is currently no effective therapy for advanced or metastatic UBC 2. Therefore, it is imperative to advance our understanding of the intricate molecular mechanisms that drives the progression and recurrence of UBC, as it could pave the way for novel therapeutic options and potentially enhance patient outcomes.

Tumor environment is regarded as a key determinant of tumor progression, especially for the plasticity and heterogeneity of cancer stem-like cells (CSCs). Within the tumor stroma, macrophages can constitute up to 50% of the cell population in certain solid tumors 3. As such, tumor-associated macrophages (TAMs) are pivotal in mediating tumor-stroma interactions, thereby promoting cancer growth, immune evasion, and recurrence 4-6. On the other hand, CSC (a cancer cell subpopulation within the tumor) has the capability to repopulate the entire tumor bulk 7. While several signaling pathways, including those driven by STAT3, Hedgehog/Gli1, and Wnt/β-catenin, are known to be active in bladder CSCs, the sustenance of their stem-like properties is intricately regulated by a combination of intrinsic signaling cascades and extrinsic cues from tumor microenvironment (TME) and the broader tumor macroenvironment (TMaE) 8-12. Hence, targeting the complex crosstalk within TME, such as the one between CSCs and macrophages presents a promising therapeutic strategy that could simultaneously impact cancer stemness and enhance the efficacy of cancer treatments.

Ubiquitin-mediated protein degradation is a crucial mechanism that significantly influences cancer progression 13. SPOP, an E3 ubiquitin ligase, has been implicated in the regulation of the T cell immune response 14. In prostate and endometrial cancers, SPOP functions as a tumor suppressor by destabilizing oncoproteins such as AR, ERG, Nanog and BRD2-4 15-19, with point mutations in its MATH domain, a region critical for enzyme-substrate interaction 20. In contrast, in kidney cancer, SPOP is considered as an oncogene because it facilitates the degradation of the tumor suppressor PTEN 21. The duality underscores the context-dependent roles of SPOP in cancers. Nevertheless, its function in UBC remains largely unknown.

In this study, we identified SPOP downregulation, which was associated with TAM infiltration in human UBC samples, promoted UBC cell proliferation and stemness, induced by macrophage. Furthermore, STAT3 served as a novel substrate for SPOP protein in UBC cells, with SPOP downregulation resulting in the elevated STAT3 and CCL2 levels. The secretion of CCL2 by UBC cells and IL-6 by macrophages within the TME acted in concert to augment tumor stemness and promote UBC progression. Finally, we demonstrated that the deficiency of transcription factor VEZF1 was a key factor driving the downregulation of SPOP in UBC patients.

## Methods

### Data acquisition

The gene expression profile datasets of UBC patients were downloaded from The Cancer Genome Atlas (TCGA-BLCA; https://portal.gdc.cancer.gov/) and from the Gene Expression Omnibus (GEO) database (http://www.ncbi.nlm.nih.gov/geo) for GSE13507, GSE31684, GSE32894, GSE48075, GSE48276, GSE87304 and GSE128702. The mRNA expression data from TCGA-BLCA were converted to FPKM (fragments per kilobase of transcript per million mapped fragments) values.

### Human UBC specimens

UBC and the adjacent normal bladder tissues (n = 48), plus tissue microarray containing another 94 UBC samples (Table S1) were obtained from Shanghai Pudong Hospital, Fudan University Pudong Medical Center, and Daping Hospital, Army Medical University, respectively. Human tissues collection in this study had obtained the approvals of Ethics Committees of Shanghai Pudong Hospital, Fudan University Pudong Medical Center, and Ethics Committees of Daping Hospital, Army Medical University, respectively. All participants provided the informed consent.

### Reverse transcription-quantitative polymerase chain reaction (RT-qPCR)

Total RNA was isolated by TRIzol (#15596018; Invitrogen, USA) and cDNA was synthesized using Prime-Script RT-PCR kit (RR047; Takara, China). qPCR was performed using SYBR qPCR Master Mix (High Rox, Q341-02; Vazyme, China). PCR primers were listed in Table S2 and *ACTB* was used as an internal control.

### Western blotting, co-immunoprecipitation (co-IP) and in vitro ubiquitination analyses

Modified RIPA buffer (25 mM Tris^.^HCl pH 7.6, 150 mM NaCl, 1% NP-40, 0.1% SDS) and NP-40 lysis buffer (P0013F; Beyotime, China), supplemented with protease and phosphatase inhibitors, were used for cell lysis in Western blotting and co-IP assays, respectively. For co-IP assay, cells were pretreated with MG132 (10 μM, S2619; Selleck, USA) for 6 h before harvest. After centrifugation, the supernatant was incubated with the indicated antibodies (Table S3) at 4℃ overnight, followed by the addition of Protein A/G PLUS-Agarose (sc-2003; Santa Cruz Biotechnology, USA) at 4℃ for 2 h. The agarose beads were washed five times with cell lysis buffer, and the precipitated proteins were subjected to Western blotting analysis. For *in vitro* ubiquitination assay, 293T cells were co-transfected with Flag-SPOP, HA-STAT3 and Myc-Ub plasmids. 6 h prior to cell harvest, the proteasome inhibitor MG132 (10 μM, Selleck) was added to the medium. HA antibody was used for co-IP and Myc antibody (Table S3) was used to detect ubiquitination level.

Equal amounts of protein from cell lysates were subjected to SDS-PAGE and transferred to PVDF membranes (Merck Millipore, USA). The membranes were immunoblotted with specific primary antibodies plus HRP-conjugated secondary antibodies (Table S3), and visualized by Super Signal West Pico Stable Peroxide Solution (180-5001E; ThermoFisher Scientific, USA).

### Immunohistochemistry (IHC) staining

IHC staining was performed using SPOP, FLAG, Ki67, CK5, F4/80, CD206, CD68 and VEZF1 antibodies (Table S3) on paraffin sections from human UBC tissues and T24 xenografts, respectively. Sodium citrate buffer (10 mM, pH 6.0) was used for antigen retrieval. The expression levels of SPOP and VEZF1 proteins were scored as the intensity value (0 = no staining, 1 = weak, 2 = moderate, and 3 = intense) multiplied by the positive-ratio value (1 = 0-25%, 2 = 26-49%, 3 = 51-75%, and 4 = 76-100%). The expression levels of CD68 and CD206 were scored by counting the number of positive cells in each field. The median IHC scores of SPOP, CD68 and CD206 were selected as a threshold for defining high and low expression, while the upper quartile for IHC scores of VEZF1 were regarded as high expression.

### Cell culture and chemicals

SV-HUC-1, T24, 5637, SW780, UMUC-3, SCaBER, J82, U937 and 293T cells were obtained from Cell Bank of Type Culture Collection of Chinese Academy of Sciences (Shanghai, China). SV-HUC-1, T24, 5637, SW780, UMUC-3, SCaBER, J82 and U937 cells were maintained in RPMI 1640 medium (Invitrogen) with 10% fetal bovine serum (FBS; Hyclone, USA); while 293T cells were cultured in DMEM (Invitrogen) with 10% FBS.

To generate the stable overexpression/knockdown cell lines, 293T cells were transfected with three plasmids (psPAX2, pMD2.G and lentiviral plasmid of interest) using Lipofectamine 2000 (#11668019; ThermoFisher Scientific) to produce lentiviral particles. Stattic (20 μM, HY-13818; MedChemExpress, USA), RS 504393 (2 μM, HY-15418; MedChemExpress), IL-6 (50 ng/ml, #200-6; PeproTech, USA) and neutralizing IL-6 antibody (100 ng/ml, MAB2061; R&D systems, USA) (Table S3) were used for cell function assays.

### Plasmids construction

pCMV10-3×FLAG-SPOP, pCDH-3×FLAG-SPOP-T2A (puromycin), pCS2-1×Myc-Ub, pCS2-4×HA-STAT3 and pCDH-3×FLAG-VEZF1 (puromycin) were constructed using PrimeSTAR MAX DNA Polymerase (R045A; Takara) and ClonExpress MultiS One Step Cloning Kit (C113-02; Vazyme). The pCS2-4×HA-STAT3-mutants were also constructed using the Mut Express II Fast Mutagenesis Kit V2 (C214-01; Vazyme). pLKO.1-shSPOP-1 (puromycin), pLKO.1-shSPOP-2 (puromycin), and pLKO.1-shSTAT3 (blasticidin) were constructed to knockdown the expression of SPOP or STAT3. The primer sequences for plasmid construction were listed in Table S2.

### Cell proliferation assay

2000 UBC cells were seeded to 96-well plate. 12 h after cell seeding was defined as the starting point. As for the non-contact co-culture system, UBC cells and U937 cells (ratio 1:1) were cultured in the bottom and upper chamber of 6-well transwell plate (0.4 μm pore size, #3450; Corning, USA) for 96 h before seeding into 96-well plate. 10 μl 3-(4,5-dimethylthiazol-2-yl)-2,5-diphenyltetrazolium bromide (MTT, 5 mg/ml, M2128; Sigma, USA) was added to each well and removed after 3 h-incubation at 37℃. Then, 100 μl dimethyl sulfoxide (DMSO, D2650; Sigma) was added into each well and incubated for 15 min. Absorbance at 490 nm was examined using a microplate reader (BioTek Instruments, USA).

### Sphere formation assay

A total of 2×10^4^ mCherry-labeled UBC cells and U937 cells (1: 1) were seeded in the 6-well ultra-low attachment plates (#3471; Corning). The cells were maintained in DMEM/F12 medium (Invitrogen) with 10 ng/ml human recombinant bFGF (100-18B; PeproTech) and 10 ng/ml EGF (AF-100-15; PeproTech). After 6 or 10 days, spheres were photographed and the spheres with the diameter greater than 50 µm were counted.

### The* in vivo* tumorigenicity assay

All animal experimental procedures were approved by the Institutional Animal Care & Use Committee of Shanghai Institute of Materia Medica, Chinese Academy of Sciences. Five-week-old male athymic nude mice (HFK Bioscience, China) were randomly categorized into two groups (n = 6 per group). A total of 5×10^6^ T24-CV and T24-SPOP cells were resuspended in 100 μl PBS:Matrigel (1:1) and subcutaneously injected into the dorsal flank of nude mouse, respectively. Tumor size was measured every 3 days for 4 weeks. The calculation formula of tumor volume in mice is: 0.5×length×width^2^. At the endpoint, xenografts were harvested for photograph, weighed, and subjected to Western blotting and IHC analyses.

### Immunofluorescence (IF) staining

Paraffin-embedded UBC and xenograft tissue sections were de-paraffinized and blocked by 1% bovine serum albumin. After sections were incubated at 4℃ overnight with primary antibodies against FLAG, CK5, F4/80, and CD206, Alexa 488, 555, and 647-conjugated secondary antibodies were added at room temperature for 1 h, respectively. 4,6-diamidino-2-phenylindole (DAPI, #28718-90-3; BBI Life Science, China) was used for counterstaining and fluorescent images were captured using Inverted Zeiss LSM880 laser scanning confocal microscope (Zeiss, Germany). Antibodies for IF staining were listed in Table S3.

### RNA-seq

Total RNAs of SPOP knockdown and control 5637 cells were isolated by TRIzol. Then, paired-end RNA-seq sequencing library was constructed and sequenced with Illumina NovaSeq 6000 sequencer (2×150 bp read length) by Majorbio Bio-pharm Biotechnology (China).

### Differential expression analysis, functional enrichment and gene set enrichment analysis (GSEA)

To identify differentially-expressed genes (DEGs) between 5637 shNC and shSPOP cells, expression level of each gene was calculated according to the transcripts per million reads (TPM) method. RSEM (http://deweylab.biostat.wisc.edu/rsem/) was used to quantify gene abundances. Differential expression analysis was performed using the Limma, and genes with |Fold change| ≥ 1.50 and p < 0.05 were considered as DEGs. In addition, KEGG and GO enrichment analyses were performed using the online tool (https://david.ncifcrf.gov/summary.jsp) with p < 0.05 compared with the whole-transcriptome background. GSEA analysis was performed on the AZARE gene set from the Broad Institute (version 4.1.0). The nominal p < 0.05 and false discovery rate (FDR) q < 0.25 were used as cutoff values.

### RNA interference

siRNAs targeting human *CUL3*, *RBX1*, *SPOP*, and *VEZF1* and non-specific control siRNA were purchased from GenePharma (Shanghai, China). Lipofectamine 2000 was used for siRNA transfection into mammalian cells, according to the manufacturer's instructions. The siRNA sequences were listed in Table S2.

### Cycloheximide (CHX) chase assay

Following treatment with the protein synthesis inhibitor CHX (100 μg/ml, #66-81-9; Sigma), cells were harvested at various timepoints. The whole cell lysate was resolved by SDS-PAGE and the levels of the proteins of interest were detected by Western blotting analysis. The band signals were semi-quantified using ImageJ software, with the 0-h timepoint as the reference.

### Enzyme-linked immunosorbent assay (ELISA)

CCL2 and IL-6 levels in culture medium from co-culture system were measured by Human CCL2/MCP-1 ELISA kit (EK1872; MultiSciences (Lianke) Biotech, Hangzhou, China) and Human IL-6 ELISA kit (EH004-96; ExCell Bio, Shanghai, China), normalized to the medium volumes.

### Macrophage migration assay

A total of 1× 10^4^ U937 cells were seeded in the upper chamber of a transwell plate (8 μm pore size, #3422; Corning), containing 150 μl of medium supplemented with 2% FBS. In the bottom chamber, 500 μl of full conditional medium (CM) from T24 (CV and SPOP) or 5637 (shNC and shSPOP) cells was added. After 8 h-incubation period, U937 cells in the upper chamber were collected, counted and calculated the cells penetrating to the bottom chamber.

### Luciferase assay

Luciferase activity was examined by a luciferase assay system (E1501; Promega, USA), according to the manufacturer's protocol. Briefly, 293T cells in 24-well plates were transfected with pCMV-3×FLAG-VEZF1, wild-type and mutant SPOP promoter-driven firefly luciferase reporters cloned into the pGL3-Basic vector, in the specified combination. The cells were harvested 48 h after transfection, and lysed to measure luciferase activity using a microplate reader (BioTek Instruments). The total protein was used for normalization.

### Chromatin immunoprecipitation (ChIP) assay

ChIP assay was performed according to the protocol of EZ-ChIP kit (#17-295; Millipore). Briefly, 1×10^6^ cells on a 10 cm dish were treated with formaldehyde to crosslink histones and DNA, followed by the sonication to shear genomic DNA into fragments ranging from 200 to 500 bp. VEZF1 antibody (2 μg) was incubated with the chromatin-protein complex at 4ºC overnight. Normal rabbit IgG antibody was used as a negative control. Subsequently, protein A agarose was added for 1 h to capture the antibody-protein complex, followed by 5 M NaCl crosslinks reversion and DNA extraction for qPCR analysis. The primers and antibodies used in ChIP assay were listed in Table S2 and Table S3.

### Statistical analysis

Statistical analysis was conducted using GraphPad Prisms 8.0 software. Data were presented as means ± SD (standard deviation), with at least three independent experiments. Student's *t* test was used to compare the differences between two groups. Pearson correlation was used to determine the correlation of different gene expressions. The log-rank (Mantel-Cox) test was applied to compare the survival distributions of the two groups in Kaplan-Meier survival analysis. A p-value less than 0.05 was considered to indicate statistically significance.

### Data availability

The RNA-seq data were available in GEO database with the number GSE240670.

## Results

### The levels of SPOP negatively correlated with UBC stage/grade and poor outcome

**Figure 1:**
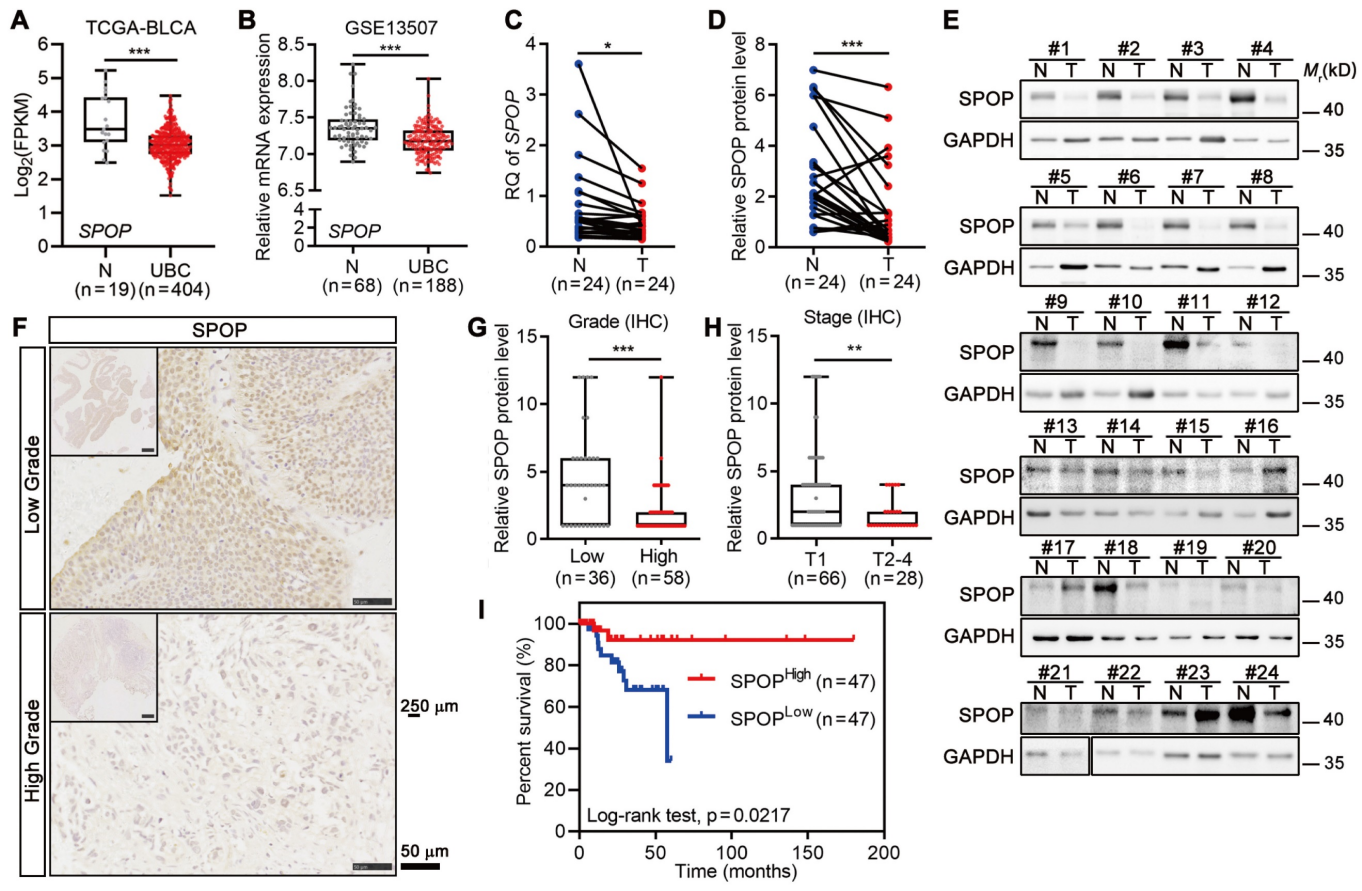
** SPOP level was negatively correlated with tumor grade, stage and clinical outcome in UBC patients. A**-**B,** Analysis of SPOP mRNA levels in public UBC datasets: TCGA-BLCA (**A**) and GSE13507 (**B**). **C,** Quantitative assessment of *SPOP* mRNA levels in 24 paired UBC (T) and adjacent normal (N) tissues from our cohort. **D**-**E,** The quantification (**D**) of Western blotting analysis (**E**) of SPOP protein levels in 24 paired UBC and adjacent normal tissues. **F,** Representative IHC staining for SPOP in low- and high-grade UBC tissues. Scale bar, 50 μm; the inset, 250 μm. **G**-**H,** SPOP protein levels by IHC in 94 UBC specimens, stratified by tumor grade (**G**) and tumor stage (**H**) from our cohort. **I,** Kaplan-Meier survival plot depicting cumulative overall survival of 94 UBC patients, stratified by SPOP protein levels using IHC staining scores. Data were shown as mean ± SD and analyzed by unpaired *t* test in (**A**-**B**, and **G-H**) or by paired *t* test in (**C**-**D**). *, p < 0.05; **, p < 0.01; ***, p < 0.001.

To examine the role of SPOP in UBC, we analyzed the frequency of *SPOP* genetic alterations in TCGA-BLCA dataset containing 127 samples with genetic information, and found that only 2.4% of UBC patients had genetic alterations, including X27 splicing and E210Q (within the BTB domain; Figure S1A-B). This suggested that *SPOP* genetic alterations were unlikely to play a major role in bladder carcinogenesis. Notably, the *SPOP* mRNA levels were significantly lower in UBC specimens compared to normal bladder tissues in both TCGA-BLCA and GSE13507 datasets (Figure 1A-B). Next, RT-qPCR and Western blotting data indicated that both SPOP mRNA and protein levels were significantly reduced in the cancerous tissues compared to their adjacent normal tissues of 24 UBC patients from our own cohort (Figure 1C-E). IHC staining on tissue microarray containing another 94 human UBC tissues from our own cohort further revealed that SPOP levels were markedly lower in high-grade tumors compared to low-grade tumors (Figure 1F-G, Table S1). A similar trend was observed when comparing high-stage tumors with low-stage tumors (Figure 1H). Notably, reduced SPOP levels in UBC patients were associated with poor clinical outcomes (Figure 1I). These findings highlighted an inverse correlation between SPOP levels and UBC aggressiveness, suggesting its potential as a prognostic biomarker for UBC patients.

### SPOP repressed UBC progression by inhibiting TAM infiltration

**Figure 2:**
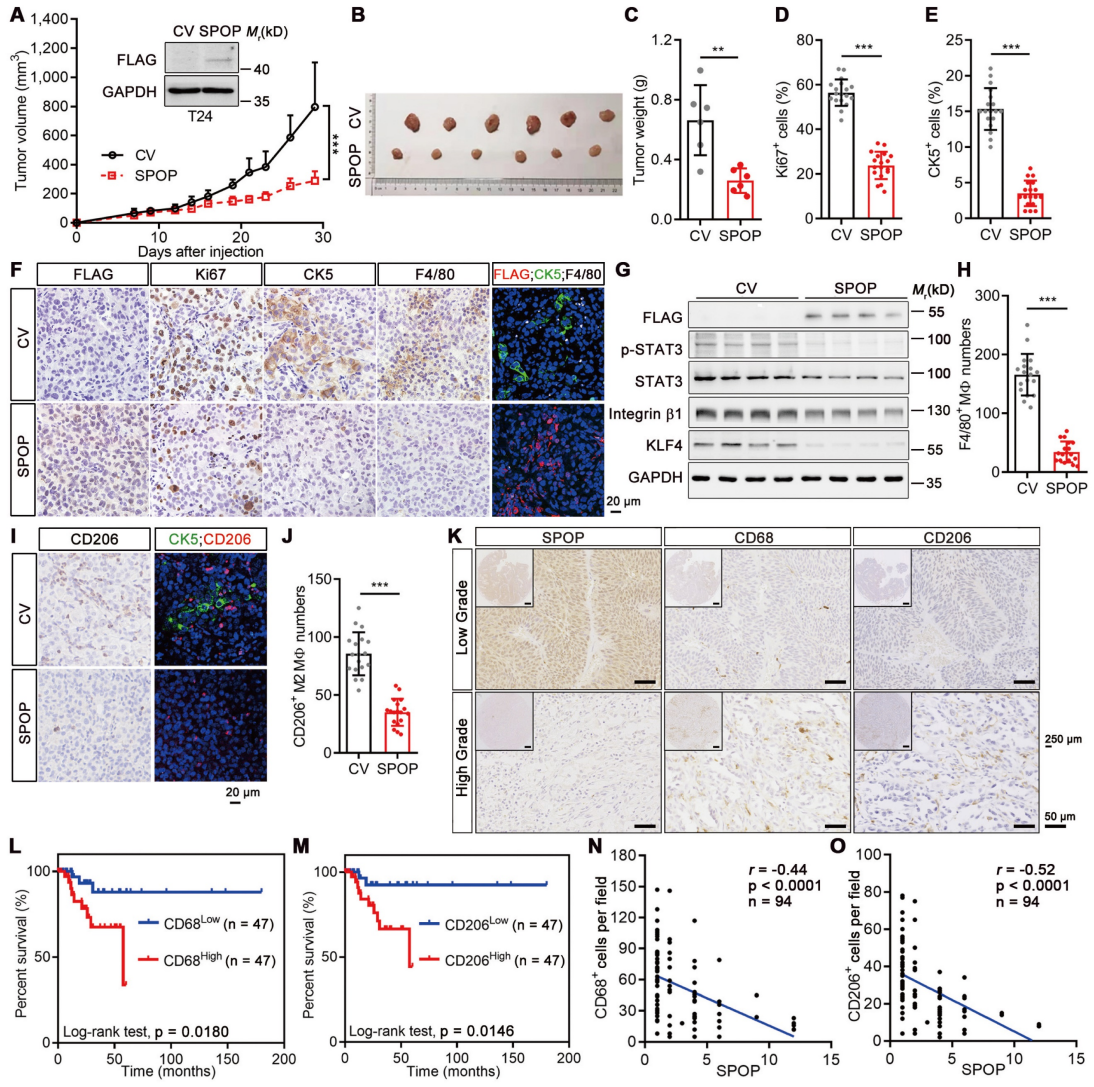
** SPOP overexpression suppressed UBC growth, stemness and TAM infiltration. A-C,** SPOP overexpression inhibited T24 xenograft growth. The growth curves (**A**), photograph (**B**) and weights (**C**) of T24 xenografts with SPOP overexpression (SPOP) and control (CV) were shown. n = 6 mice per group. The ectopic expression of SPOP in T24 cells was confirmed by Western blotting analysis (**A**, inset). **D-F,** Comparisons of the proliferative (Ki67^+^, **D**) and cancer stemness (CK5^+^, **E**) indices between SPOP and CV groups, with representative IHC and IF staining data (**F**). FLAG-tagged SPOP (red), CK5 (green) and F4/80 (white) with DAPI counterstaining (blue) were shown in **F**. Scale bar, 20 μm. **G**, Western blotting analysis of stemness markers in T24-CV and -SPOP xenografts. **H**-**J**, Comparative assessment of macrophage (F4/80^+^, **H**) and M2-like macrophage (CD206^+^, **I** and **J**) number in SPOP and CV groups, with representative IHC and IF staining data shown in **F** and **I**. Scale bar, 20 μm. **K,** Representative IHC staining for SPOP, CD68 and CD206 in human low-grade (upper panels) and high-grade (lower panels) UBC tissues. Scale bar, 50 μm; the inset, 250 μm. **L**-**M,** Kaplan-Meier survival plots for 94 UBC patients, stratified by CD68^+^ (**L**) and CD206^+^ (**M**) macrophage numbers, according to the IHC staining data, showing cumulative overall survival. **N**-**O**, Correlations between SPOP levels and CD68^+^ (**N**) and CD206^+^ (**O**) cell number/field in 94 UBC specimens, as determined by IHC staining. Data were shown as mean ± SD and analyzed by unpaired *t* test in (**A**, **C-E**, **H** and **J**). r, Pearson correlation coefficient. **, p < 0.01; ***, p < 0.001.

Next, we compared the SPOP levels across various urothelium-derived cell lines and found that non-malignant SV-HUC-1 and 5637 UBC cells exhibited higher SPOP levels compared to UMUC-3 and T24 UBC cells (Figure S1C). To elucidate the role of SPOP in UBC cells, FLAG-tagged SPOP was ectopically expressed in T24 cells (Figure 2A, inset). However, SPOP overexpression influenced neither cell proliferation nor sphere formation (Figure S1D-F). Interestingly, it suppressed xenograft growth, evidenced by decreasing tumor volumes and weights (Figure 2A-C). SPOP overexpression led to a significant reduction in the number of Ki67-positive (a proliferation marker) UBC cells and CK5-positive (a bladder CSC marker) CSCs within the xenografts (Figure 2D-F). It also reduced the expression of cancer stemness markers, including phosphorylated STAT3(Y705), Integrin β1 and KLF4 (Figure 2G). The discrepancy between the *in vitro* and *in vivo* data hinted at the possible influence of extrinsic factors from the TME in mediating the tumor-suppressive effects of SPOP.

Given that macrophage is one of the most influential subpopulations within tumor stroma 3, we conducted IHC staining against the macrophage marker F4/80 and found that the number of macrophages was significantly reduced when SPOP was overexpressed (Figure 2F, 2H). IF staining for FLAG-tagged SPOP, CK5 and F4/80 revealed that CK5-positive CSCs intertwined with TAMs in the control xenografts. SPOP overexpression significantly diminished both CK5^+^ CSCs (Figure 2E-F; right IF panels) and the presence of total and tumor-promoting M2 macrophages, as indicated by the reduced F4/80^+^ (Figure 2F, 2H) and CD206^+^ cell number (Figure 2I-J). To further validate these findings, we conducted IHC on human UBC tissues using antibodies for CD68 (a pan-macrophage marker) and CD206 (an M2 macrophage marker; Figure 2K). The infiltration of macrophage and M2 macrophage could predict poor prognosis in UBC patients, respectively (Figure 2L-M). SPOP levels also showed a significantly inverse correlation with the number of CD68^+^ and CD206^+^ macrophages in UBCs (Figure 2N-O). Collectively, these data demonstrated that SPOP repressed UBC stemness by inhibiting TAM infiltration.

### The role of macrophage in SPOP-repressed UBC cell proliferation and stemness

**Figure 3:**
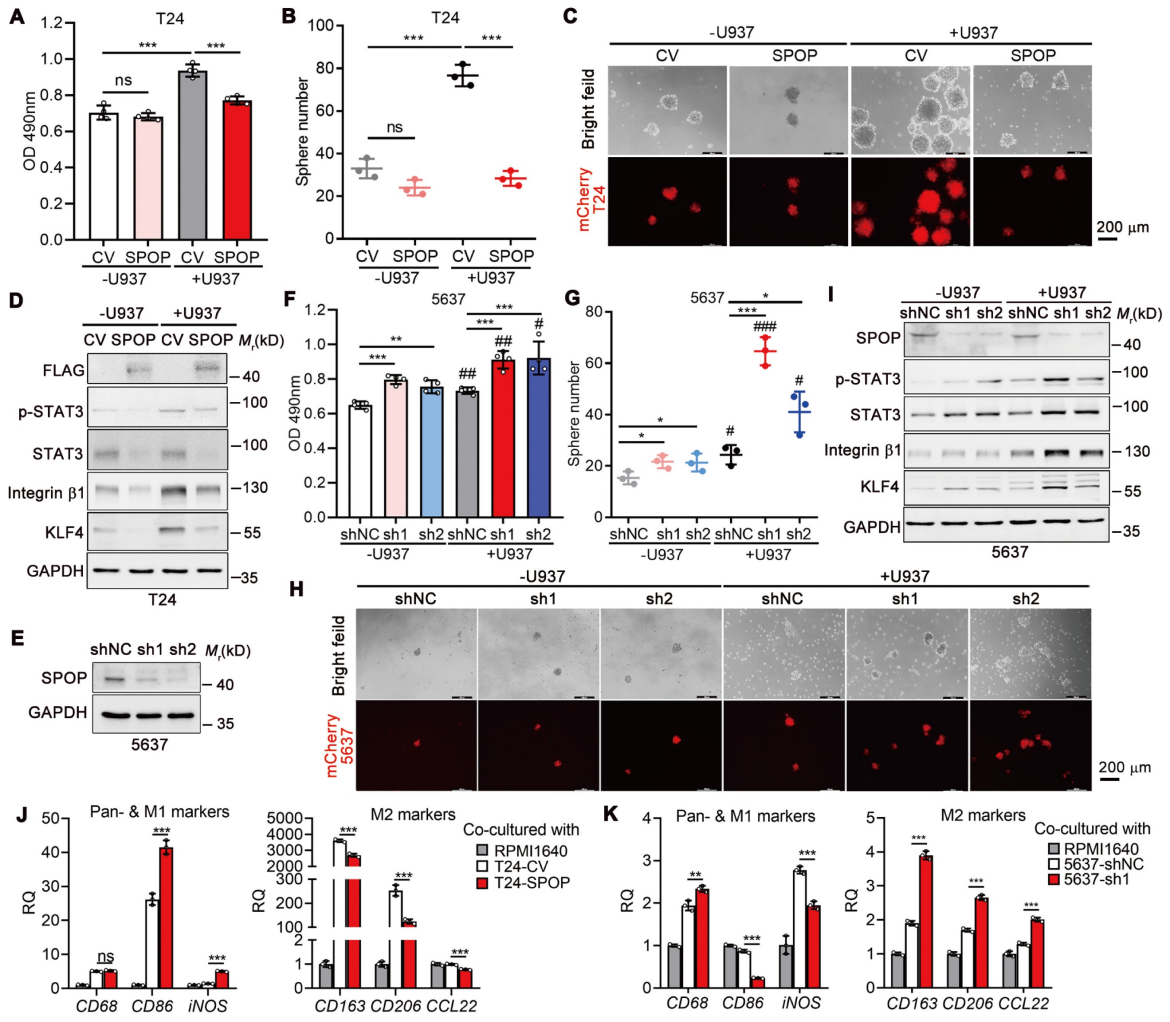
** Interactional regulation between UBC cells and macrophages in cancer proliferation and stemness. A,** MTT assay on T24 cells (CV and SPOP) after co-cultured with or without U937 cells in a non-contact system for 4 days. **B**-**C,** Quantification (**B**) and representative images (**C**) of mCherry-labeled T24 spheres (CV and SPOP) co-cultured with or without U937 cells for 6 days. Scale bar, 200 μm. **D,** Western blotting analysis of stemness markers in T24 cells (CV and SPOP) co-cultured with or without U937 cells in a non-contact system for 4 days. **E,** Stable knockdown of SPOP in 5637 cells using two different shRNAs (sh1 and sh2) by Western blotting analysis. shNC represented the negative control. **F,** MTT assay on control and two SPOP knockdown 5637 cells for 3 days, after co-cultured with or without U937 cells in a non-contact system for 4 days.** G**-**H,** Quantification (**G**) and representative images (**H**) of mCherry-labelled control and SPOP knockdown 5637 spheres co-cultured with and without U937 cells for 10 days. Scale bar, 200 μm. **I,** Western blotting analysis of stemness markers in 5637 cells (shNC, sh1 and sh2) co-cultured with or without U937 cells in a non-contact system for 4 days. **J**-**K,** RT-qPCR analysis of U937 cells co-cultured in a non-contact way with T24 cells (CV and SPOP; **J**) and 5637 cells (shNC and sh1; **K**) to assess expression of pan-marker (*CD68*), M1-like marker (*CD86* and *iNOS*), and M2-like markers (*CD163, CD206* and *CCL22*) in macrophages. Cell culture medium alone (RPMI1640) served as the negative control. Data were presented as mean ± SD and analyzed by unpaired *t* test. *, p < 0.05; **, p < 0.01; ***, p < 0.001; ns, p ≥ 0.05. #, p < 0.05; ##, p < 0.01; ###, p < 0.001; with *vs* without co-cultured U937 cells.

To establish the suppressive role of SPOP on macrophage-mediated UBC cell proliferation and cancer stemness, we performed co-culture experiments with T24 and U937 cells, a human monocytic cell line capable of differentiating into macrophage, in a non-contact setting. In the absence of U937 cells, there was no significant difference in the proliferation rate between T24 cells overexpressing SPOP and control cells; however, in the presence of U937 cells, ectopic SPOP expression significantly inhibited U937-induced UBC cell proliferation (Figure 3A). Meanwhile, sphere formation assays indicated that U937 cells led to a significant increase in both the number and size of mCherry-labelled T24 spheres, whereas SPOP overexpression effectively mitigated this U937-induced enhancement (Figure 3B-C). The data were further confirmed by the changes in the levels of stemness markers by SPOP overexpression (Figure 3D).

Conversely, depletion of SPOP in 5637 cells with high endogenous SPOP levels by two distinct shRNAs (sh1 and sh2; Figure 3E) led to modest yet significant increases in proliferation, sphere formation, and the expression of stemness markers compared to control cells. These effects, attributed to SPOP regulation, were significantly amplified when the cells were co-cultured with U937 cells (Figure 3F-I). Similar results were observed in another UBC cell line, SW780 (Figure S2). Given the established importance of infiltrated macrophages in the suppression of UBC cell proliferation and stemness by SPOP, and considering the opposing roles of M1-like and M2-like macrophages in tumor growth 22, we assessed the mRNA levels of macrophage biomarkers, including the pan-marker (*CD68*), M1 marker (*CD86* and *iNOS*), and M2 markers (*CD163*, *CD206* and *CCL22*) in U937 cells co-cultured with UBC cells. Our results indicated that SPOP overexpression in T24 cells promoted the differentiation of U937 cells towards an M1-like phenotype, as evidenced by elevated *CD86* and *iNOS* expression and reduced levels of *CD163, CD206* and *CCL22* (Figure 3J), while SPOP knockdown in 5637 cells promoted the differentiation of U937 cells towards an M2-like phenotype (Figure 3K). These findings implied a complex crosstalk between UBC cells and infiltrated macrophages.

### SPOP enhanced ubiquitination-dependent STAT3 degradation in UBC cells

**Figure 4:**
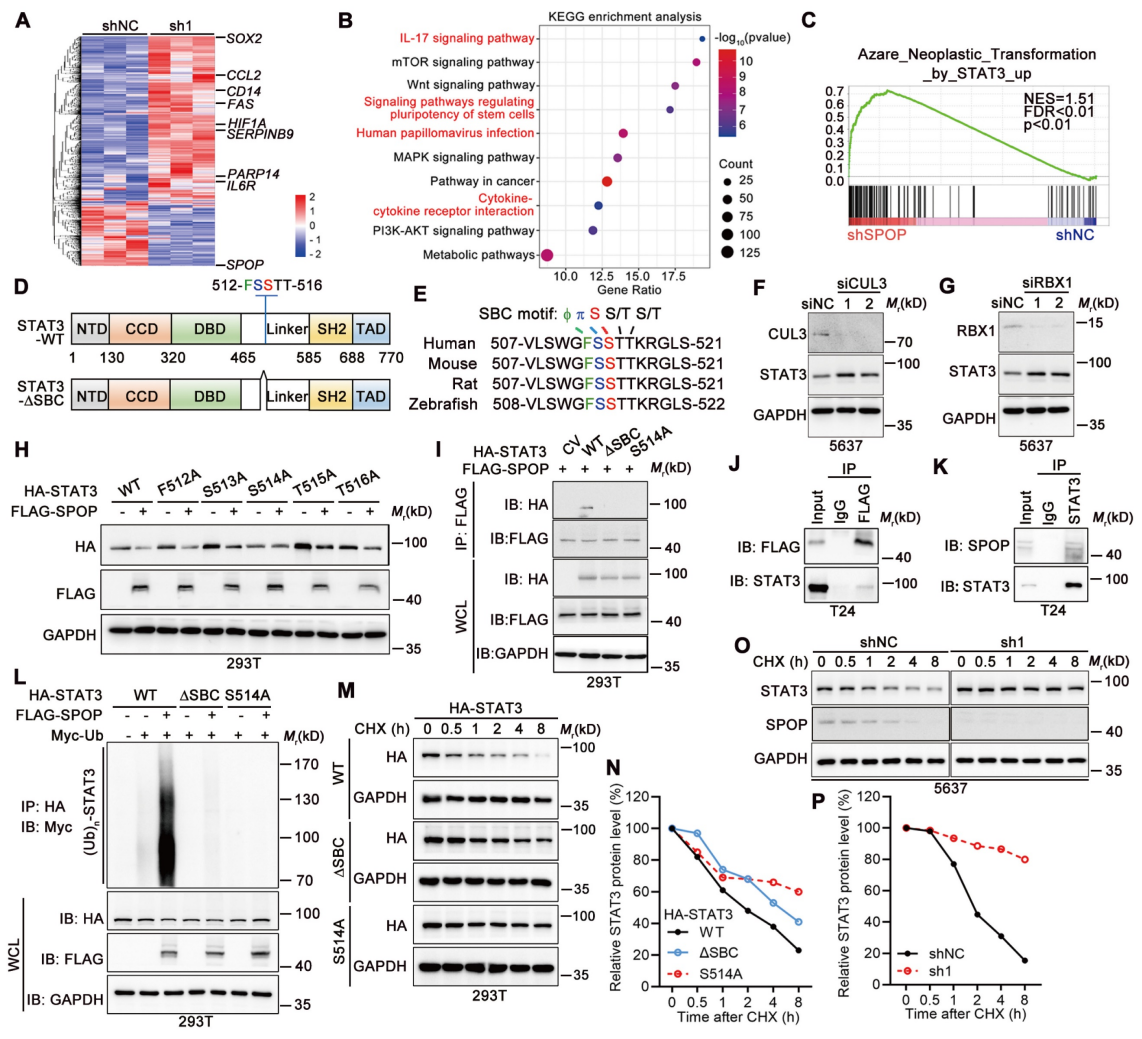
** SPOP mediated the degradation of STAT3 protein. A,** Heatmap displaying DEGs between SPOP knockdown (sh1) and control (shNC) 5637 cells as determined by RNA-seq (|Fold change| ≥ 1.50; p < 0.05). **B**, KEGG enrichment analysis identified the pathways affected by SPOP knockdown in 5637 cells. **C**, GSEA analysis on the RNA-seq data. **D**, The structure of STAT3 protein, indicating the location of a putative SBC motif. **E**, Sequence alignment of STAT3 protein across different species, highlighting the presence of a putative SBC motif with conserved nonpolar (Φ) and polar (π) residues, as well as serine (S) and threonine (T) residues.** F**-**G**, Western blotting analysis of STAT3 protein levels in 5637 cells following transient transfection with siRNAs targeting CUL3 (siCLU3) (**F**) or RBX1 (siRBX1) (**G**), compared to siRNA control (siNC). **H,** Western blotting analysis of HA-tagged wild-type STAT3 and its SBC motif mutants, along with FLAG-tagged SPOP in 293T cells co-transfected with the indicated plasmids. **I,** Co-IP assay in 293T cells transiently transfected with HA-tagged STAT3 (WT, ΔSBC, and S514A) and FLAG-tagged SPOP, using a FLAG-tag antibody for IP and an HA-tag antibody to detect ectopic STAT3. WCL, whole cell lysate. **J,** Co-IP assay in T24 cells transiently transfected with FLAG-tagged SPOP, using a FLAG-tag antibody for IP and a STAT3 antibody to detect endogenous STAT3. **K,** Co-IP assay in T24 cells using a STAT3 antibody for IP and a SPOP antibody to detect endogenous SPOP. **L,** Western blotting analysis on WCL and anti-HA tag IP from 293T cells co-transfected with indicated plasmids, including HA-tagged STAT3 (WT, ΔSBC and S514A), FLAG-tagged SPOP, and Myc-tagged ubiquitin.** M**-**N,** Western blotting analysis (**M**) and quantification (**N**) for HA-STAT3 WT and its mutants (ΔSBC and S514A) protein levels in 293T cells, after treatment with 100 μg/ml cycloheximide (CHX) for the indicated timepoints. The 0-h timepoint was used as a normalization control. **O**-**P**, Western blotting analysis (**O**) and quantification (**P**) of endogenous STAT3 protein levels in SPOP-knockdown (sh1) and control (shNC) 5637 cells after CHX treatment for the indicated timepoints.

To dissect the role of SPOP in crosstalk between UBC cells and macrophages, we performed RNA-seq to identify DEGs in 5637 cells with SPOP knockdown (Figure 4A). Both KEGG and GO enrichment analyses highlighted the significant association with inflammatory response and signaling pathways involved in stem cells (Figure 4B, Figure S3A). GSEA further revealed a significant correlation between SPOP loss and STAT3 activation, which is involved in both cancer stemness and immune regulation (Figure 4C). Next, we validated the expression levels of eight STAT3 target genes 23-25, including *CCL2*, *IL6R* and *SOX2*, which were induced in 5637 cells with SPOP depletion and suppressed in T24 cells overexpressing SPOP, compared to their controls (Figure S3B-C). Given that both total and phosphorylated STAT3 levels exhibited a negative correlation with SPOP expression (Figure 2G, 3D, 3I, Figure S2E), and that STAT3 contains an evolutionarily conserved putative SPOP-binding consensus (SBC) motif, 512-FSSTT-516 (Φ-π-S-S/T-S/T; Φ, nonpolar residues; π, polar residues; S, Serine; T, Threonine; Figure 4D-E), we inferred that STAT3 may be a substrate of SPOP.

Since SPOP-mediated protein degradation relies on the SPOP/CUL3/RBX1 E3 ubiquitin ligase complex 26, depletion of either CUL3 or RBX1 resulted in STAT3 stabilization in 5637 cells (Figure 4F-G). FLAG-tagged SPOP could downregulate HA-tagged STAT3 when they were co-expressed in 293T cells. Furthermore, co-expression of SPOP and STAT3 with five different SBC motif mutants revealed that only S514A mutant within SBC motif abrogated SPOP's ability to degrade STAT3 protein (Figure 4H). Co-IP assay showed that the intact S514 of STAT3 was essential for its interaction with SPOP (Figure 4I), which was confirmed by both overexpressed and endogenous protein contexts in T24 cells (Figure 4J-K). Additionally, SPOP markedly promoted STAT3 poly-ubiquitination, an effect that was diminished when SBC motif was deleted or S514 was mutated (Figure 4L). Consistent with this line, the half-lives of STAT3-ΔSBC and STAT3-S514A mutants were found to be longer than that of wild-type STAT3 (STAT3-WT; Figure 4M-N), and SPOP depletion led to a prolonged half-life of STAT3 (Figure 4O-P). Altogether, these results demonstrated that serine residue at position 514 within SBC motif of STAT3 protein was essential for its degradation mediated by SPOP.

### SPOP repressed macrophage-mediated UBC cell proliferation and cancer stemness via STAT3 downregulation

**Figure 5:**
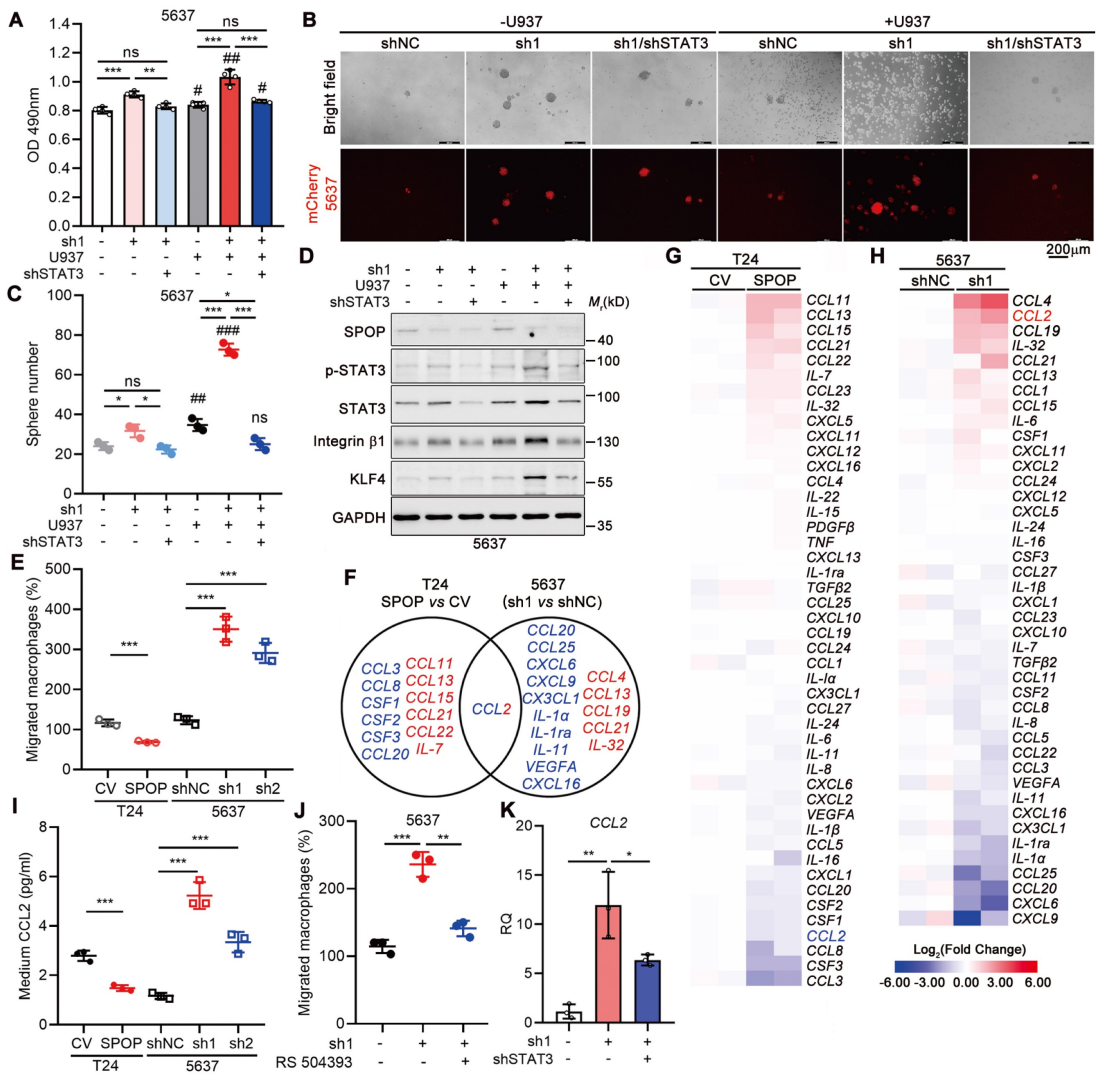
**STAT3 inhibition by SPOP diminished macrophage-mediated UBC cell proliferation and stemness. A,** MTT assay on 5637 cells (shNC, sh1, and sh1+shSTAT3), after 4-day co-culture with or without U937 cells in a non-contact system. **B**-**C**, Representative images (**B**) and quantification (**C**) of mCherry-labelled 5637 spheres (shNC, sh1, and sh1+shSTAT3) after 10-day co-culture with or without U937 cells. Scale bar, 200 μm.** D,** Western blotting analysis of stemness markers in 5637 cells (shNC, sh1, and sh1+shSTAT3) co-cultured with or without U937 cells. **E,** Quantification of U937 cell migration in a transwell system in response to the CM from T24 cells (CV and SPOP) and 5637 cells (shNC, sh1, and sh2). **F-H,** A Venn diagram (**F**) illustrated the RT-qPCR array for 48 cytokines/chemokines in T24 cells (CV and SPOP) (**G**) and 5637 cells (shNC and sh1) (**H**). The undetected genes, such as *CXCL9* and *IL-33* in T24 cells and *CXCL13*, *IL-15*,* IL-22*,* IL-33*, *PDGFβ* and *TNF* in 5637 cells, were not shown. Blue, downregulated genes; red, upregulated genes. **I,** ELISA for CCL2 secretion in the CM of T24 cells (CV and SPOP) and 5637 cells (shNC, sh1, and sh2). **J,** Quantification of U937 cell migration in a transwell system in response to the CM from 5637 cells (shNC, sh1, and sh1 treated with RS 504393 (2 μM), a CCL2 receptor inhibitor). **K,** *CCL2* mRNA levels in 5637 cells (shNC, sh1, and sh1+shSTAT3) by RT-qPCR assay. sh1 or sh2, SPOP-knockdown; shNC, shRNA control. Data were presented as mean ± SD and analyzed by unpaired *t* test. *, p < 0.05; **, p < 0.01; ***, p < 0.001; ns, p ≥ 0.05. #, p < 0.05; ##, p < 0.01; ###, p < 0.001; with *vs* without co-cultured U937 cells.

To elucidate the contribution of STAT3 in SPOP depletion-induced cancer progression, we knocked down STAT3 in 5637 cells lacking SPOP. Our findings indicated that both SPOP deletion and co-culture with U937 cells enhanced cancer cell proliferation and sphere formation, with a synergistic effect observed when these two conditions were combined. STAT3 knockdown substantially reversed these effects (Figure 5A-C), which was further confirmed by the reduced expression of stemness markers (Figure 5D). Consistent with the impact of STAT3 knockdown, treatment of UBC cells with Stattic, a specific small molecule inhibitor targeting STAT3, similarly inhibited cell proliferation and sphere formation (Figure S3D-E). These results supported the notion that SPOP inhibited macrophage-mediated UBC cell proliferation and stemness by negatively regulating STAT3 activation.

### SPOP targeted STAT3/CCL2 axis in UBC cells to repress macrophage infiltration

Given the negative correlation between SPOP expression and macrophage infiltration in human specimens and mouse xenografts, we investigated the role of SPOP in modulating macrophage recruitment in UBC. Utilizing a transwell assay, we assessed macrophage migration in response to co-culture with UBC cells. Our findings indicated a significant reduction in macrophage migration when macrophages were exposed to the CM from T24 cells overexpressing SPOP (Figure 5E). Conversely, the migration of macrophages was significantly enhanced when cultured in the CM from 5637 cells with SPOP depletion (Figure 5E).

To uncover the cytokines/chemokines in the CM that may mediate the effect of SPOP on macrophage migration, we conducted an RT-qPCR array of 48 genes implicated in cell-cell crosstalk within the TME 11, 27-28. This analysis revealed seven cytokines/chemokines were downregulated in T24 cells with SPOP overexpression and six were upregulated in SPOP-depleted 5637 cells, respectively (Figure 5F-H). CCL2 was the only chemokine significantly altered in both assays (Figure 5F), which was corroborated by ELISA (Figure 5I). Moreover, the CCR2 inhibitor RS 504393 29 significantly repressed the macrophage migration induced by SPOP knockdown (Figure 5J). Because *CCL2* mRNA level was increased upon SPOP knockdown in 5637 cells and this increase was partially reversed by STAT3 depletion (Figure 5K), we proposed that SPOP inhibited macrophage infiltration by disrupting the STAT3/CCL2 signaling axis.

### SPOP inhibited UBC cell proliferation and cancer stemness by repressing macrophage-secreted IL-6

**Figure 6:**
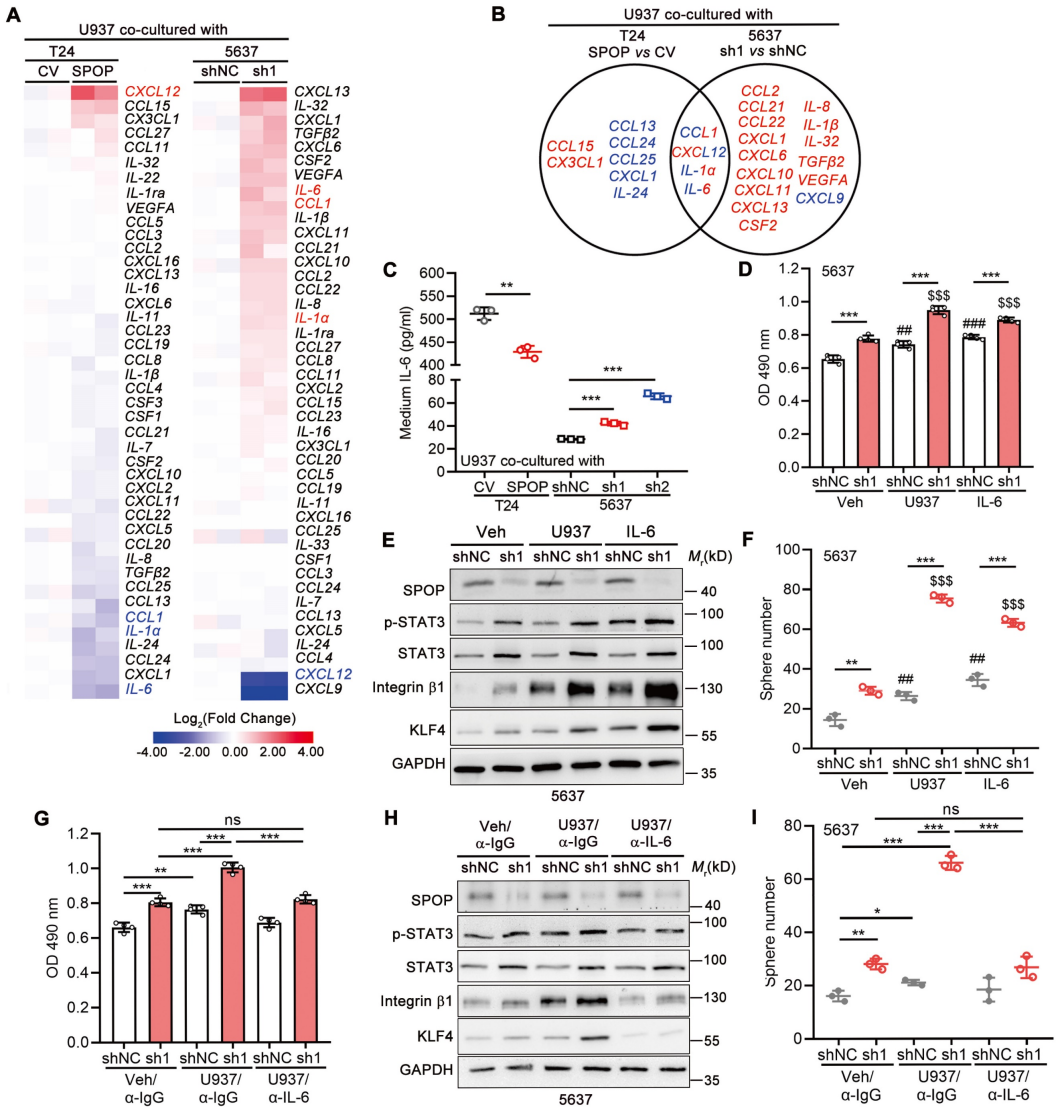
** Macrophage-secreted IL-6 enhanced UBC cell proliferation and cancer stemness, which was negatively regulated by SPOP. A,** The heatmaps of RT-qPCR arrays for 48 cytokines/chemokines in U937 cells co-cultured with T24 cells (CV and SPOP; left panel) or 5637 cells (shNC and sh1; right panel). The undetectable genes (*CXCL9*, *IL-15, IL-33*, *PDGFβ*, and *TNF* in T24 cells and *CSF3*, *IL-15*,* IL-22*,* PDGFβ*, and *TNF* in 5637 cells) were not shown. **B,** A Venn diagram of macrophage-derived chemokine/cytokine genes, mediated by SPOP in UBC cells. Blue, downregulated genes; red, upregulated genes. **C**, ELISA for IL-6 secretion from U937 cells co-cultured with T24 cells (CV and SPOP), as well as 5637 cells (shNC, sh1 and sh2). **D-F**, Assessment of UBC cell proliferation and stemness by MTT assay (**D**), Western blotting analysis for stemness marker protein levels (**E**), and quantification of mCherry-labelled 5637 spheres (**F**) between 5637 shNC and sh1 cells, co-cultured with or without U937 cells or treated with IL-6 (50 ng/ml) for 4 days.** ##**, p < 0.01; **###**, p < 0.001; or $$$, p < 0.001; co-culture with U937 or treatment of IL-6 *vs* vehicle control. **G-I,** Assessment of UBC cell proliferation and stemness by MTT assay (**G**), Western blotting analysis for stemness marker protein levels (**H**), and quantification of mCherry-labelled 5637 spheres (**I**) between 5637 shNC and sh1, co-cultured with or without U937 cells for 4 days. The addition of α-IL-6 neutralizing antibody (100 ng/ml) or isotype control IgG in the presence of U937 cells. sh1, SPOP-knockdown; shNC, shRNA control. Data were presented as mean ± SD and analyzed by unpaired *t* test. *, p < 0.05; **, p < 0.01; ***, p < 0.001; ns, p ≥ 0.05.

We further explored the effects of SPOP in UBC cells on cytokine/chemokine expression in macrophage. As shown in Figure 6A, eight cytokines/chemokines were downregulated (≤ 0.67-fold) in U937 cells co-cultured with T24 cells overexpressing SPOP, whereas seventeen cytokines/chemokines were upregulated (≥ 1.50-fold) in U937 cells co-cultured with SPOP-depleted 5637 cells, with IL-6 being the most remarkably changed gene in both scenarios (Figure 6B). In line with the RT-qPCR array data, ELISA for IL-6 showed that SPOP overexpression in T24 cells inhibited IL-6 secretion from U937 cells, while SPOP depletion in 5637 cells promoted IL-6 secretion from U937 cells (Figure 6C). Moreover, IL-6 significantly enhanced cell proliferation and cancer stemness, while such effects were exacerbated by SPOP knockdown in 5637 cells (Figure 6D-F). The U937-induced cancer aggressiveness were abrogated by the administration of an IL-6 neutralizing antibody (Figure 6G-I). Conversely, SPOP overexpression significantly attenuated IL-6-driven enhancements in cancer aggressiveness in T24 cells (Figure S3F-G). Above data indicated that SPOP depletion promoted the secretion of macrophage-derived IL-6 into TME, thereby contributing to the escalation of UBC cell proliferation and the induction of cancer stemness.

### VEZF1 transcriptionally upregulated SPOP in UBC cells

**Figure 7:**
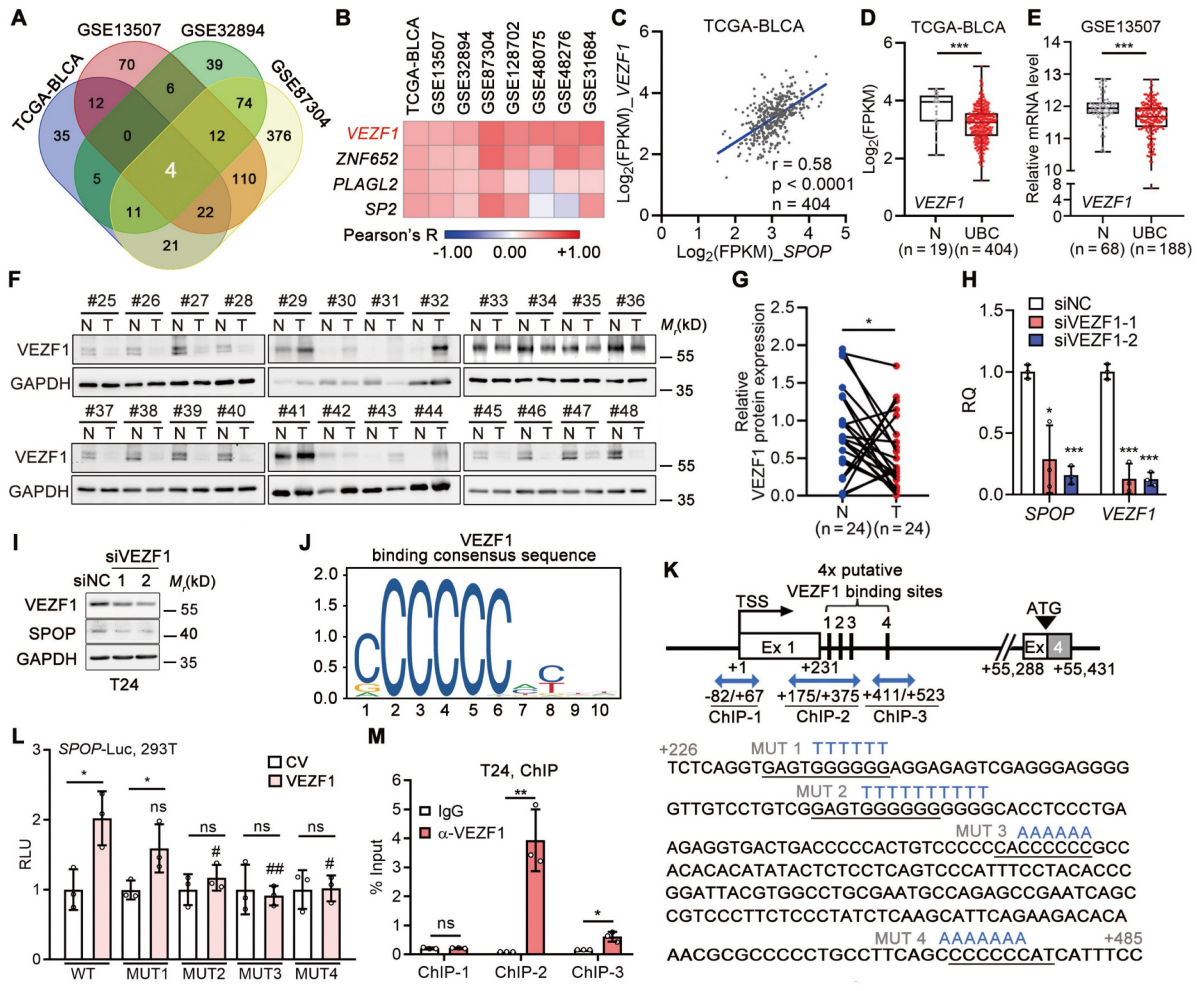
** SPOP was a direct downstream target of VEZF1 in UBC cells. A**, A Venn diagram illustrating a significantly positive association between transcriptional factors from Human TFome library and *SPOP* mRNA level in four UBC datasets. **B**, Heatmap depicting the correlations between four transcriptional factors and *SPOP* mRNA level in eight UBC public datasets, determined by Pearson correlation analysis. **C**, Pearson correlation of *SPOP* and *VEZF1* mRNA levels in TCGA-BLCA dataset. **D**-**E**, *VEZF1* mRNA levels in normal bladder and UBC samples from TCGA-BLCA (**D**) and GSE13507 (**E**) datasets. **F**-**G**, Western blotting analysis (**F**) and quantification (**G**) of SPOP protein levels in 24 paired UBC (T) and adjacent normal (N) tissues. **H**-**I**, The depletion of VEZF1 reduced SPOP at mRNA (**H**) and protein level (**I**) in T24 cells. **J**, VEZF1 binding consensus sequence from JASPAR database. **K**, Schematic representation of four putative VEZF1 binding sites within intron 1 of *SPOP* gene, predicted by JASPAR. Three amplicons for ChIP assay were indicated by blue arrow, with mutation sequences for each binding site marked in blue. Ex, exon. **L**, Luciferase activity assay on different SPOP reporters, containing wild-type (WT) or four mutant VEZF1 binding sites (MUT) following VEZF1 overexpression in 293T cells. CV, control vector. **M**, ChIP assay by VEZF1 antibody on *SPOP* gene promoter in T24 cells, with IgG as a negative control. Data were shown as mean ± SD and analyzed by unpaired *t* test in (**D**-**E, H, L-M**) or by paired *t* test in (**G**). *, p < 0.05; **, p < 0.01; ***, p < 0.001; ns, p ≥ 0.05. #, p < 0.05; ##, p < 0.01; MUT *vs* WT-reporter luciferase activity with VEZF1 overexpression.

In light of the frequent downregulation, rather than mutation, of *SPOP* gene in UBC patients, we explored the relationships between *SPOP* and 1,564 transcriptional factors within the Human TFome library 30 across four datasets (n = 1,205 in total; Figure 7A, Figure S4A). Our analysis revealed a positive correlation between *SPOP* and *VEZF1* (Figure 7B-C), an endothelial cell-specific transcription factor 31, which was further confirmed in another four independent datasets (Figure S4B-H). In TCGA-BLCA and GSE13507 datasets, *VEZF1* mRNA was significantly downregulated in UBC samples (Figure 7D-E), and VEZF1 protein was lower in another 24 UBC tissues than the paired normal ones (Figure 7F-G). VEZF1 knockdown led to a significant reduction in SPOP mRNA and protein levels (Figure 7H-I); meanwhile, VEZF1 overexpression resulted in SPOP upregulation in a concentration-dependent manner (Figure S4I). To ascertain whether VEZF1 can regulate SPOP transcription, we identified four potential VEZF1 binding site within *SPOP* gene intron 1 using JAPSPAR database (http://jaspar.genereg.net/; Figure 7J-K). Wild-type luciferase reporter containing this evolutionarily conserved region (-122 to +705 bp; Figure S4J), and four mutated reporters (with mutations on each VEZF1 binding site) were constructed (Figure 7K). Luciferase assay indicated that three sites (MUT2-4) were critical for VEZF1 binding (Figure 7L), which was confirmed by ChIP assay (Figure 7M). These data indicated that SPOP was a VEZF1 target gene.

### VEZF1 reduced the macrophage-mediated UBC aggressiveness

**Figure 8:**
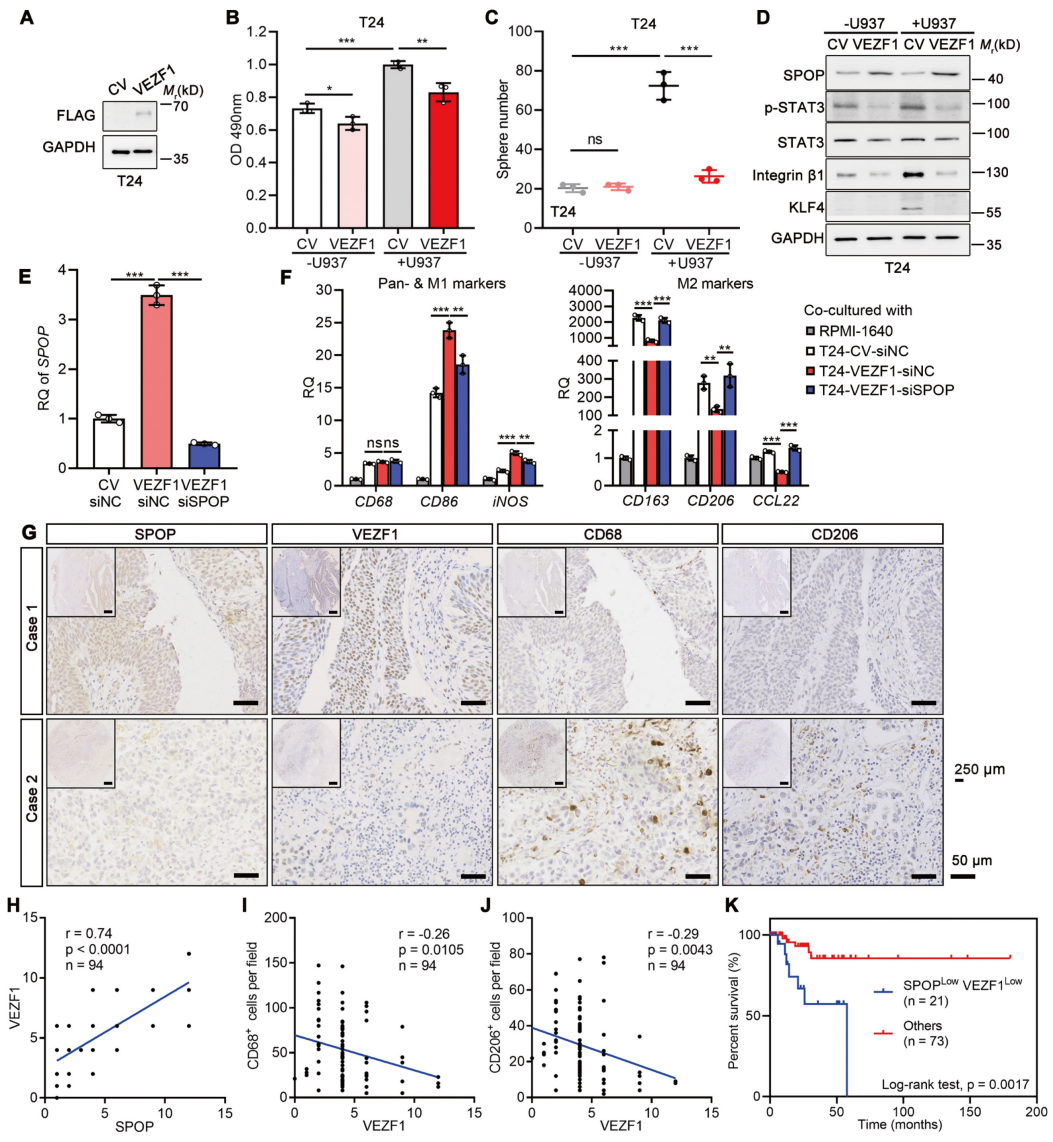
** VEZF1 suppressed macrophage-mediated UBC proliferation and stemness. A,** Western blotting analysis confirming the ectopic expression of VEZF1 in T24 cells.** B,** MTT assay on T24 cells (CV and VEZF1) after 96 h co-cultured with or without U937 cells in a non-contact system. **C,** Sphere assay performed on mCherry-labelled T24 cells (CV and VEZF1) co-cultured with or without U937 cells at day 6. **D,** Western blotting analysis for stemness markers in T24 cells with VEZF1 overexpression co-cultured with or without U937 cells.** E,** SPOP depletion in T24 cells with VEZF1 overexpression using siRNA (siSPOP) by RT-qPCR analysis. siNC, negative control. **F,** RT-qPCR analysis on U937 cells co-cultured in a non-contact way with T24 cells with VEZF1 overexpression (VEZF1; CV, negative control)/SPOP depletion (siSPOP; siNC, negative control) to assess expression of pan-marker (*CD68*), M1-like marker (*CD86* and *iNOS*), and M2-like markers (*CD163*, *CD206* and *CCL22*) in macrophages. Cell culture medium alone (RPMI1640) served as the negative control. **G**, Expression of SPOP, VEZF1, CD68 and CD206 in 94 human UBC tissues by IHC staining. Representative images from two UBC patients were displayed. Scale bar, 50 μm; the inset, 250 μm. **H-J**, The association of VEZF1 and SPOP protein levels (**H**), CD68^+^ macrophage number (**I**) and CD206^+^ macrophage number (**J**) in 94 human UBC patients. **K,** Kaplan-Meier survival plot of cumulative overall survival of 94 UBC patients, divided by SPOP^Low^VEZF1^Low^ *vs* Others. Data were presented as mean ± SD and analyzed by unpaired *t* test. *, p < 0.05; **, p < 0.01; ***, p < 0.001; ns, p ≥ 0.05.

Overexpressed VEZF1 in T24 cells significantly counteracted the proliferation and cancer stemness induced by U937 cells (Figure 8A-C). The effects were accompanied by upregulation of SPOP and downregulation of cancer stemness markers (Figure 8D). To explore whether VEZF1 overexpression could modify the M1/M2 polarization of macrophages via SPOP, U937 cells co-cultured with T24 cells with VEZF1 overexpression (VEZF1-siNC) and SPOP depletion by siRNA (VEZF1-siSPOP). As Figure 8E-F shown, VEZF1 overexpression alone upregulated SPOP expression, induced the M1-like polarization (p < 0.001), and suppressed the M2-like polarization (p < 0.01). Consistently, SPOP depletion in T24 cells with VEZF1 overexpression reversed the above effects significantly (p < 0.01). These results suggested that VEZF1 overexpression in UBC cells could induce the M2 to M1-like polarization in macrophages co-cultured with UBC cells, which was mainly dependent on SPOP upregulation. IHC staining on the tissue array containing 94 human UBC patients revealed nuclear localization of VEZF1 in UBC cells (Figure 8G). VEZF1 protein in UBC patients showed a significant positive correlation with SPOP protein expression (Figure 8H), and significant negative correlations with the number of CD68^+^ and CD206^+^ macrophages (Figure 8I-J) or lymph node metastasis (N stage; Table S1). Low *VEZF1* expression also exhibited a poorer prognosis in UBCs (Figure S4K). Consistent with the findings from the GSE48075 dataset (Figure S4L-Q), patients with low levels of both SPOP and VEZF1 exhibited significantly worse overall survival outcomes (Figure 8K).

## Discussion

The key regulatory networks, which orchestrate cancer stemness maintenance and the interaction of CSCs with TME, are crucial for cancer development and recurrence. In this study, we have identified that SPOP, which is frequently downregulated in UBC patients, plays a suppressive role in regulating cancer stemness and immunosuppressive TME. SPOP deficiency led to the STAT3 protein stabilization and the increased CCL2 expression and secretion, thereby facilitating macrophage recruitment and polarization. In a feedback loop, the polarized macrophages secreted IL-6, which further activated STAT3 signaling in UBC cells, thus maintaining cancer stemness. Additionally, SPOP was a transcription factor VEZF1 target gene, and the co-downregulation of VEZF1 and SPOP predicted a worse clinical outcome in UBC patients. Together, we have delineated the tumor-suppressive function of SPOP in UBC, demonstrating that it restricts bladder CSC characteristics and TAM recruitment and polarization by targeting the STAT3 oncoprotein for degradation.

Unlike prostate and endometrial cancers, SPOP in UBC is not commonly altered at the genetic level, but is subject to other regulatory influences. Recent studies have highlighted the negative regulation of SPOP by miRNAs, C/EBP-α, TGF-β/SMAD signaling and promoter hypermethylation in gastric, lung, prostate and colorectal cancers, respectively 32-36. We uncovered that SPOP was a direct transcriptional target of the VEZF1 protein, which is historically recognized as a vascular endothelium-specific transcription factor 31. Recently VEZF1 exhibited overexpression in breast and liver cancers, suggesting a potential role in promoting cancer progression 37-39. However, in the context of UBC, VEZF1 appears to exert a protective effect, counteracting the aggressive behavior induced by macrophages in cancer cells through the induction of SPOP.

The sustained STAT3 activation has been linked to the acquisition of CSC properties and the promotion of carcinogen-induced UBC progression 9,40. In addition to the phosphorylation on STAT3 nuclear translocation and transcriptional activity, STAT3 can also be intricately modulated via proteasome pathway under various conditions, including mumps virus infection and hyperosmotic stress 41,42. In activated B-cell-like diffuse large B-cell lymphoma and breast cancer cells, two E3 ligases, Fbw7 and MARCH8, were reported to degrade STAT3, respectively 43,44. As a novel SPOP substrate, STAT3 drives a battery of genes involved in cancer stemness maintenance, including SOX2. Besides, Nanog is also a direct STAT3 target gene to contribute to self-renewal of liver CSC 45. Recent studies have also highlighted the role of SPOP in governing the stability of Nanog, a stemness marker, in prostate cancer 18,19. Therefore, SPOP may regulate cancer stemness through multiple dimensions in a cancer content-dependent manner. Frequent downregulation of SPOP in UBC cells resulted in STAT3 protein stabilization, thereby endowing UBC cells with CSC characteristics, which may represent an intrinsic driver of tumor aggressiveness.

The plasticity between CSCs and non-CSCs populations is intricately modulated by extrinsic cues derived from TME 3,46. A significant correlation between an elevated cancer stemness signature and a constrained immune response has been observed across 21 solid cancers, suggesting an interplay between CSC population and immune cells 47. In line with this notion, macrophages within the TME created a tumor-supportive niche, promoting cancer stemness through paracrine or juxtacrine pathways, which in turn, to augment tumor chemoresistance and metastatic potential 47-49. Furthermore, glioma stem cells recruited monocyte-derived macrophages by secreting CSCs-derived periostin, thereby supporting tumor growth 50; additionally, the loss of PTEN and NF1 in glioma stem cells upregulated LOX and chemokines, respectively, to attract TAMs 51,52. In our experimental model, UBC cells with SPOP deficiency impeded STAT3 degradation, bolstered CCL2 expression and secretion, and facilitated macrophage recruitment. Reciprocally, these polarized macrophages augmented IL-6 secretion, which in turn activated STAT3 through its receptor IL-6R, further promoting the CSC properties in UBC cells as a potential extrinsic driving force.

Our study elucidates the intricate interaction between SPOP-deficient CSCs and TAMs in UBC via the CCL2-IL-6 cytokine axis. Given that high macrophages infiltration correlates with a poor prognosis in UBC patients, disrupting this detrimental CSC-macrophage cycle represents a promising therapeutic strategy. In fact, clinical trials have explored the potential of CCR2/5-inhibitors, such as BMS-813160, in combination with neoadjuvant nivolumab for non-small cell lung cancer or liver cancer, currently in a phase II trial (NCT04123379) 53. Additionally, IL-6 receptor inhibitors, including Sarilumab (NCT05428007) and Tocilizumab (NCT03999749), have been evaluated in combination with Ipilimumab and Nivolumab for patients with unresectable stage III or stage IV melanoma in separate phase II studies 54,55.

Previous literatures reported that loss-of-function mutations in SPOP compromised ubiquitination-mediated PD-L1 degradation, leading to increased PD-L1 levels and reduced numbers of tumor-infiltrating lymphocytes in mouse tumors and in primary human prostate cancer specimens 14. On the other hand, TAMs, as tumor-infiltrating lymphocytes, do express PD-1 on the surface, which was associated with disease stage of human cancers 56,57; PD-1^+^ TAMs reduced the phagocytic capacity to eliminate PD-L1^+^ cancer cells, compared with PD-L1^-^ cancer cells 56. Based on these results, we may speculate that loss-of-expression in SPOP might also stabilize PD-L1 protein in bladder cancer cells and induce immune escape via TAMs, beyond the STAT3/CCL2/IL-6 axis. Further studies should be performed to investigate this hypothesis.

## Conclusions

Our data delineate the presence of a VEZF1/SPOP/STAT3 regulatory axis within bladder CSCs that orchestrates the establishment of CSC-to-macrophage crosstalk to maintain cancer stemness and immunosuppressive TME, underscoring its clinical significance in UBC prognosis and highlighting its potential as a therapeutic target.

## Supplementary Material

Supplementary figures and tables.

## Abbreviations

UBC: urothelial bladder cancer
CSC: cancer stem-like cells
TAM: tumor-associated macrophage
TME: tumor microenvironment
TMaE: tumor macroenvironment
TCGA: The Cancer Genome Atlas
GEO: Gene Expression Omnibus
FPKM: fragments per kilobase of transcript per million mapped fragments
RT-qPCR: reverse transcription-quantitative polymerase chain reaction
IP: immunoprecipitation
IHC: immunohistochemistry
MTT: 3-(4,5-dimethylthiazol-2-yl)-2,5-diphenyltetrazolium bromide
DMSO: dimethyl sulfoxide
IF: immunofluorescence
DAPI: 4,6-diamidino-2-phenylindole
GSEA: gene set enrichment analysis
DEG: differentially-expressed gene
TPM: transcripts per million reads
FDR: false discovery rate
CHX: cycloheximide
ELISA: enzyme-linked immunosorbent assay
CM: conditional medium
ChIP: chromatin immunoprecipitation
SD: standard deviation
SBC: SPOP-binding consensus
