# Supplementary material for: SPOP downregulation promotes bladder cancer progression based on cancer cell-macrophage crosstalk via STAT3/CCL2/IL-6 axis and is regulated by VEZF1
Source: Theranostics. 2024 Oct 7;14(17):6543–59. doi: 10.7150/thno.101575 (PMC11519788; doi:10.7150/thno.101575)
Supplement: Supplementary file 1 — Supplementary figures and tables. [file thnov14p6543s1.pdf]

Supplementary Information

SPOP downregulation promotes bladder cancer progression based on cancer cell-macrophage crosstalk via STAT3/CCL2/IL-6 axis and is regulated by VEZF1

Meiqian Li*, Yangyan Cui*, Qi Qi, Jiakuan Liu, Jiaxuan Li, Guifang Huang, Jiale Yang, Jingya Sun, Zhihui Ma, Shengjie Liang, Dianzheng Zhang, Jun Jiang, Rujian Zhu[#], Qiuli Liu[#], Ruimin Huang[#], Jun Yan[#]

*These authors contributed equally to this article.

#Corresponding authors:

Jun Yan, Ph.D., Fudan University, Shanghai 200032, China. Phone: 86-21-54237454; E-mail: yan_jun@fudan.edu.cn

Ruimin Huang, Ph.D., Shanghai Institute of Materia Medica, Chinese Academy of Sciences, Shanghai 201203, China. Phone: 86-21-68077978; E-mail: rmhuang@simm.ac.cn

Qiuli Liu, M.D., Daping Hospital, Army Medical University, Chongqing 400042, China. Phone: 86-23-68729241; E-mail: liuqiuli900827@163.com

Rujian Zhu, M.D., Shanghai Pudong Hospital, Fudan University Pudong Medical Center, Shanghai, China. Phone:86-18918753975; E-mail: tzzhurj@163.com

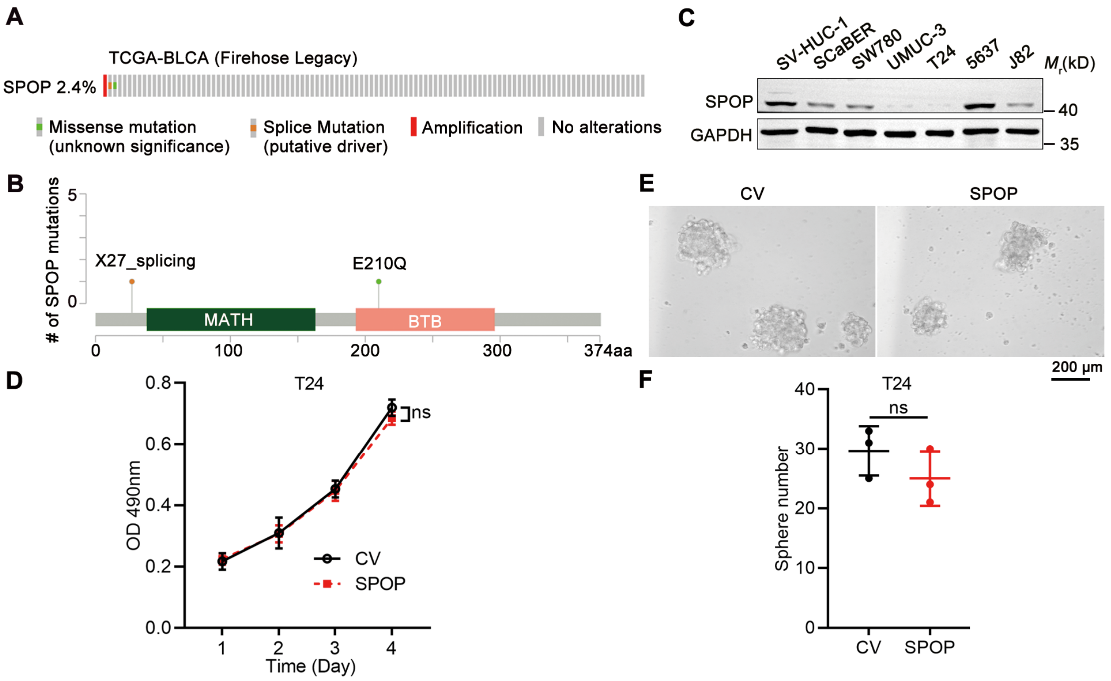

Supplementary Figure S1. Rare genetic alterations of *SPOP* gene in human UBC specimens and its function in UBC cell proliferation and cancer stemness. A, The genetic alterations of *SPOP* in 127 UBC patients with mutation information from TCGA-BLCA dataset. **B**, Two SPOP mutations were depicted on SPOP protein. **C**, Western blotting analysis on SPOP protein in non-malignant SV-HUC-1 and six UBC cell lines. **D**, MTT assay on T24 cells with SPOP overexpression (SPOP) and control cells (CV) for 4 days. **E-F**, Representative images (**E**) and quantification (**F**) of sphere number of SPOP-overexpressing (SPOP) and control (CV) T24 cells for 6 days. Scale bar, 200 μm. Data were presented as mean ± SD and analyzed by Student's *t* test. ns, $p \geq 0.05$.

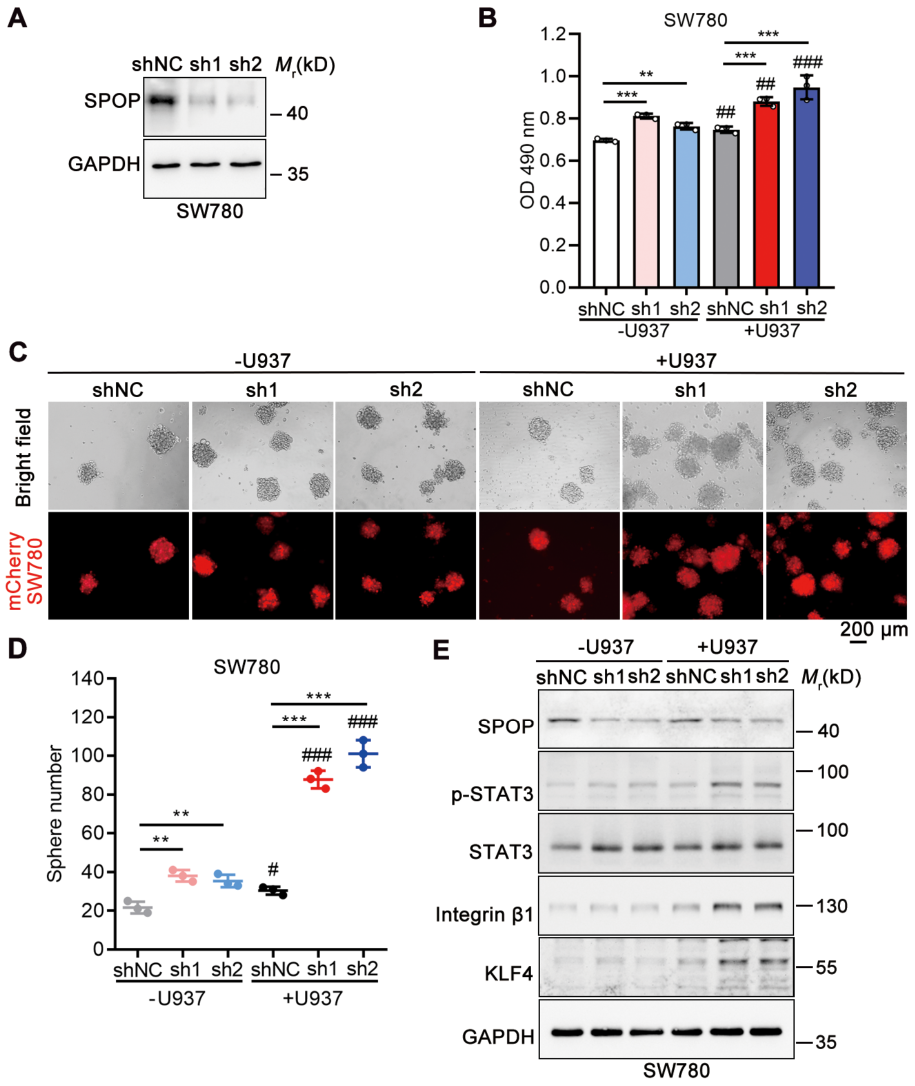

Supplementary Figure S2. SPOP suppressed macrophage-mediated proliferation and stemness in SW780 cells. A, The knockdown efficiency of SPOP in SW780 cells by RNA interfering was assessed by Western blotting analysis. **B**, MTT assay on control (shNC) and SPOP knockdown (sh1 and sh2) SW780 cells for 3 days, after co-cultured with or without U937 cells in a non-contact system. **C**-**D**, Representative images (**C**) and quantification (**D**) of sphere formation in mCherry-labelled SPOP deficient (sh1 and sh2) and control (shNC) SW780 cells, co-cultured with and without U937 cells over a 6-day period. Scale bar, 200 μm. **E**, Western blotting analysis of stemness markers in SPOP-knockdown SW780 cells, co-cultured with and without U937 cells. Data were presented as mean ± SD and analyzed by unpaired *t* test (**B&D**). **, $p < 0.01$; ***, $p < 0.001$. #, $p < 0.05$; ##, $p < 0.01$; ###, $p < 0.001$; with *vs* without co-cultured U937 cells.

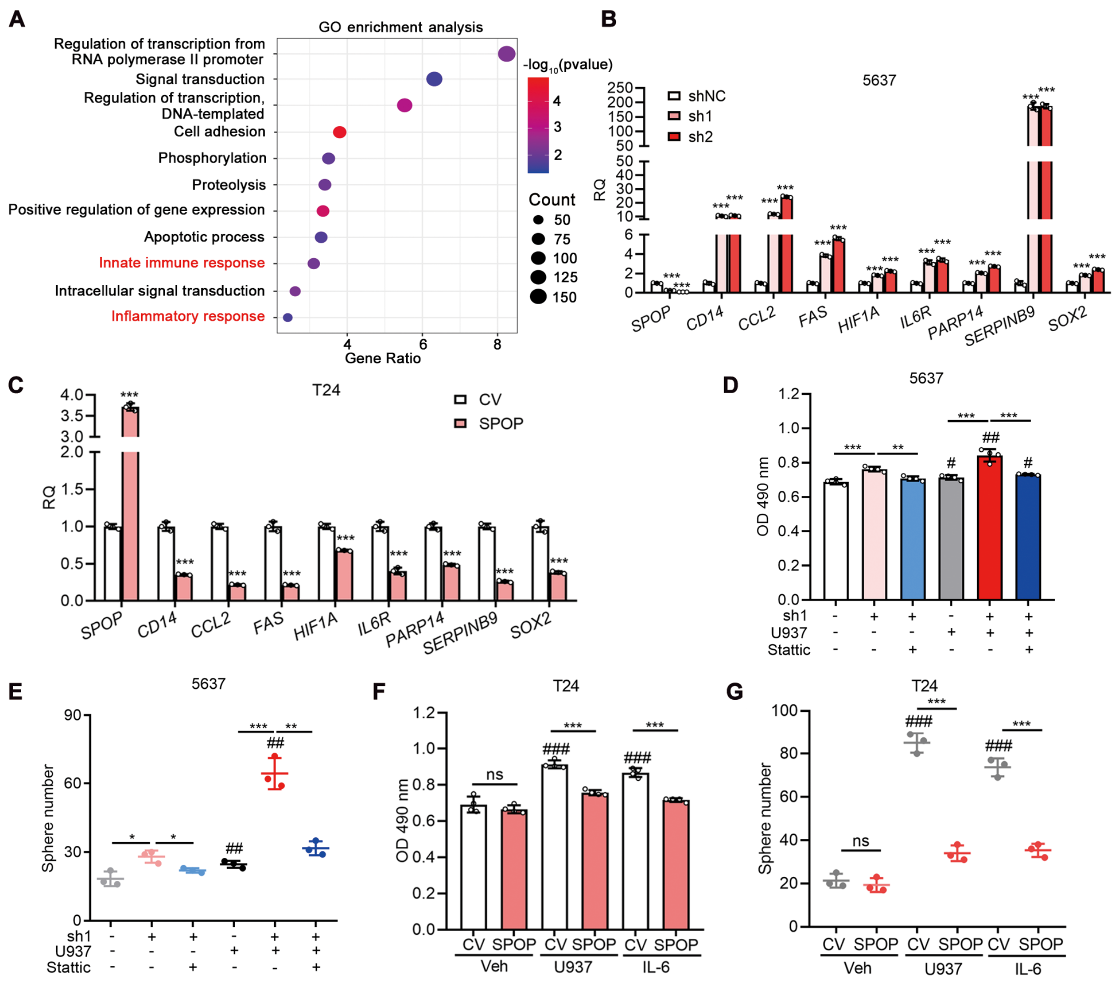

Supplementary Figure S3. STAT3 activation was required for SPOP downregulation-induced UBC cell proliferation and cancer stemness via the interaction with macrophage. A, GO enrichment analysis identified the pathways affected by SPOP knockdown in 5637 cells. **B-C**, Validation of SPOP and STAT3's target genes in SPOP knockdown (sh1 and sh2) and control (shNC) 5637 cells (**B**), as well as in SPOP-overexpressing (SPOP) and control (CV) T24 cells (**C**) by RT-qPCR analysis. **D**, MTT assay on control (shNC) and SPOP knockdown (sh1) 5637 cells, after 96 h co-cultured with or without U937 cells in a non-contact system. Stattic (20 μM) was added to block STAT3 activation. **E**, Quantification of mCherry-labelled control (shNC) and SPOP knockdown (sh1) 5637 spheres, co-cultured with and without U937 cells for 10 days. Stattic was added to block STAT3 activation. **F**, MTT assay on T24 cells (CV and SPOP) for 3 days, after co-cultured with U937 cells in a non-contact system or treated with IL-6. **G**, Quantification of sphere number of

mCherry-labelled SPOP-overexpressing (SPOP) and control (CV) T24 cells, co-cultured with or without U937 cells or treated with IL-6 (50 ng/ml) after 6-day sphere formation. Data were presented as mean ± SD and analyzed by unpaired *t* test. *, $p < 0.05$; **, $p < 0.01$; ***, $p < 0.001$; ns, $p \geq 0.05$. #, $p < 0.05$; ##, $p < 0.01$; ###, $p < 0.001$; with *vs* without co-cultured U937 cells or with *vs* without Stattic or IL-6 treatment.

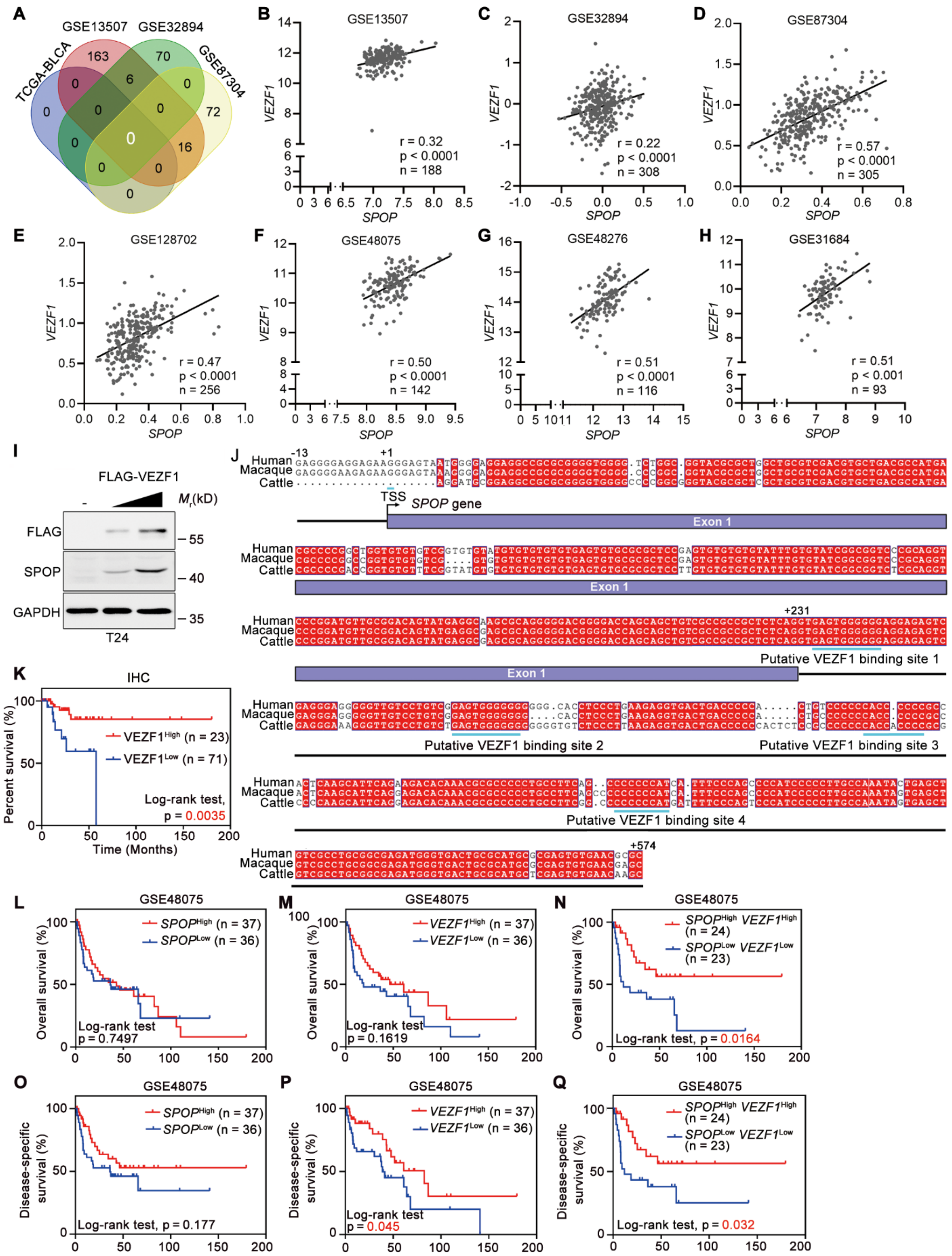

Supplementary Figure S4. The correlation between VEZF1 and SPOP in UBC patients and cells. A, A Venn diagram showing the significantly negative association between transcriptional factors derived from Human TFome library and *SPOP* mRNA levels in four UBC datasets. **B-H**, Pearson correlation analysis of *SPOP* and *VEZF1* mRNA levels in seven UBC datasets, including GSE13507 (**B**), GSE32894 (**C**),

GSE87304 (**D**), GSE128702 (**E**), GSE48075 (**F**), GSE48276 (**G**) and GSE31684 (**H**). **I**, Western blotting analysis of SPOP protein levels in T24 cells transfected with different amounts of pFLAG-VEZF1 plasmid. **J**, The alignment of evolutionarily conserved *SPOP* promoter sequences from three different species. Transcriptional start site (TSS) and four putative VEZF1 binding sites, predicted by JASPAR, were labelled underlying the nucleotide sequences. **K**, Kaplan–Meier survival plot of cumulative overall survival of 94 UBC patients from our own cohort stratified by VEZF1 protein level, according to the IHC staining score on tissue microarray. **L-N**, Kaplan–Meier survival plots of cumulative overall survival of UBC patients from GSE48075 dataset, stratified by *SPOP* mRNA level **(L)**, *VEZF1* mRNA level (**M**) and $SPOP^{Low}VEZF1^{Low}$ *vs* $SPOP^{High}VEZF1^{High}$ (**N**). **O-Q**, Kaplan–Meier survival plots of cumulative disease-specific survival of UBC patients from GSE48075 dataset, stratified by *SPOP* mRNA level (**O**), *VEZF1* mRNA level (**P**) and $SPOP^{Low}VEZF1^{Low}$ *vs* $SPOP^{High}VEZF1^{High}$ (**Q**).

Supplementary Table S1. Correlation between immunohistochemical variables and clinicopathologic features in UBC patients from our own cohort (n = 94).

Characteristics	n	Expression of SPOP High (n, %)	Expression of SPOP Low (n, %)	p	$CD68^+$ TAM High (n, %)	$CD68^+$ TAM Low (n, %)	p	$CD206^+$ TAM High (n, %)	$CD206^+$ TAM Low (n, %)	p	Expression of VEZF1 High (n, %)	Expression of VEZF1 Low (n, %)	p
**Gender**				0.1196			0.3545			**0.0271**			0.1586
**Male**	82	44 (54%)	38 (46%)		39 (48%)	43 (52%)		37 (45%)	45 (55%)		64 (78%)	18 (22%)	
**Female**	12	3 (25%)	9 (75%)		8 (67%)	4 (33%)		10 (83%)	2 (17%)		7 (58%)	5 (42%)	
**Age**				0.1438			0.8349			>0.9999			0.8101
**<65**	40	24 (60%)	16 (40%)		19 (48%)	21 (52%)		20 (50%)	20 (50%)		31 (78%)	9 (22%)	
**≥65**	54	23 (43%)	31 (57%)		28 (52%)	26 (48%)		27 (50%)	27 (50%)		40 (74%)	14 (26%)	
**Smoking**				>0.999			0.6320			0.3374			0.5772
**Yes**	23	11 (48%)	12 (52%)		10 (43%)	13 (57%)		9 (39%)	14 (61%)		16 (70%)	7 (30%)	
**No**	71	36 (51%)	35 (49%)		37 (52%)	34 (48%)		38 (%)	33 (%)		55 (77%)	16 (23%)	
**Tumor grade**				**0.019**			**0.0055**			0.1370			>0.9999
**Low**	36	24 (67%)	12 (33%)		11 (31%)	25 (69%)		14 (39%)	22 (61%)		27 (75%)	9 (25%)	
**High**	58	23 (40%)	35 (60%)		36 (62%)	22 (38%)		33 (57%)	25 (43%)		44 (76%)	14 (24%)	
**T stage**				**0.012**			**0.0414**			0.1136			0.1190
**T1**	66	39 (59%)	27 (41%)		28 (42%)	38 (58%)		29 (44%)	37 (56%)		53 (80%)	13 (20%)	
**T2-4**	28	8 (29%)	20 (71%)		19 (68%)	9 (32%)		18 (64%)	10 (36%)		18 (64%)	10 (36%)	
**N stage**				0.120			0.3545			0.1196			**0.0078**
**N0**	82	44 (54%)	38 (46%)		39 (48%)	43 (52%)		38 (46%)	44 (54%)		66 (80%)	16 (20%)	
**≥N1**	12	3 (25%)	9 (75%)		8 (67%)	4 (33%)		9 (75%)	3 (25%)		5 (42%)	7 (58%)	
**M stage**				>0.999			>0.9999			>0.9999			0.1472
**M0**	91	46 (51%)	45 (49%)		46 (51%)	45 (49%)		46 (51%)	45 (45%)		70 (77%)	21 (23%)	
**≥M1**	3	1 (33%)	2 (67%)		1 (33%)	2 (67%)		1 (33%)	2 (67%)		1 (33%)	2 (67%)	
**Tumor number**				0.2524			0.0956			0.1498			0.4443
**NA**	2	2 (100%)	0 (0%)		1 (50%)	1 (50%)		0 (0%)	2 (100%)		2 (100%)	0 (0%)	
**1**	23	13 (57%)	10 (43%)		7 (30%)	16 (70%)		9 (39%)	14 (61%)		19 (83%)	4 (17%)	
**≥1**	69	32 (46%)	37 (54%)		39 (57%)	30 (43%)		38 (55%)	33 (45%)		50 (72%)	19 (28%)	

Notes: UBC, urothelial bladder cancer. The Pearson Chi-square test was applied. Numbers in bold indicate p value with statistical difference.

Supplementary Table S2. The sequences of primers and siRNAs

Name	Forward primer (5’-3’)	Reverse primer (5’-3’)
Subcloning		
pCMV10-3×FLAG-SPOP-WT	ATTTGCGGCCGCGATGTCAAGGGTTCCAAGTCCTCC	CGGGATCCTTAGGATTGCTTCAGGCGTTTG
pCDH-3×FLAG-SPOP-WT	ACGATGACAAGCTTGCGGCCGCGATGTCAAGGGTTCCAAGTCCTCC	TCTGCCCTCGATATCGAATTCGGATTGCTTCAGGCGTTTGCG
pCS2-1×Myc-Ub	GACTTGAATCGGCCTGAATTCATGCAGATCTTCGTGAAAACCCTC	TAGTCATGAAGATCTCTCGAGTCAACCACCACGCAGGCGGAGC
pCDH-3×FLAG-VEZF1-WT	AAGCTTGCGGCCGCGAATTCAGAGGCCAACTGGACCGCGT	TTCGCGGCCGCCCGGGTTAGTTACCAAGGCGGTGATGTAG
pCS2-4×HA-STAT3-WT	TACGCTGGCCGGCCTGAATTCATGGCTCAGTGGAACCAGCTG	GCTATCTAGTCATGAAGATGTTCACATGGGGGAGGTAGCACACTC
pCS2-4×HA-STAT3-ΔSBC	CTCAGCTGGCAGAAGCGGGGGCTG	CAGCCCCCGCTCTGCCAGCTGAG
pCS2-4×HA-STAT3-F512A	AGCTGGCAGGCATCGTCCACCACCAAGCGGGGGC	GGTGGACGATGCCTGCCAGCTGAGCACCTCGGC
pCS2-4×HA-STAT3-S513A	TGGCAGTTCGCATCCACCACCAAGCGGGGGCTGAG	GGTGGTGGATGCGAACTGCCAGCTGAGCACCTCGGC
pCS2-4×HA-STAT3-S514A	CAGTTCTCGGCAACCACCAAGCGGGGGCTGAGC	CTTGGTGGTTGCCGAGAACTGCCAGCTGAGCACCTCG
pCS2-4×HA-STAT3-T515A	TTCTCGTCCGCAACCAAGCGGGGGCTGAGCATCGA	CCGCTTGGTTGCGGACGAGAACTGCCAGCTGAGCAC
pCS2-4×HA-STAT3-T516A	TCGTCCACCGCAAAGCGGGGGCTGAGCATCGAGC	CCCCCGCTTTGCGGTGGACGAGAACTGCCAGCTGAG
pLKO.1-shSPOP-1-puro	CCGGCACAAGGCTATCTTAGCAGCTCTCGAGAGCTGCTAAGATAGCCTTGTGTTTTTG	AATTCAAAAACACAAGGCTATCTTAGCAGCTCTCGAGAGCTGCTAAGATAGCCTTGTG
pLKO.1-shSPOP-2-puro	CCGGCTCCTACATGTGGACCATCAACTCGAGTTGATGGTCCACATGTAGGAGTTTTTG	AATTCAAAAACTCCTACATGTGGACCATCAACTCGAGTTGATGGTCCACATGTAGGAG
pLKO.1-shSTAT3-1-BSD	CCGGCTCAGAGGATCCCGGAAATTTCTCGAGAAATTTCCGGGATCCTCTGAGTTTTTG	AATTCAAAAACTCAGAGGATCCCGGAAATTTCTCGAGAAATTTCCGGGATCCTCTGAG
pGL3-SPOP- WT-Luciferase	TCTCTATCGATAGGTACAACAGGCTCATTATCTGG	GTACCGGAATGCCAAGCTCTCTCAAATCCCCGGGCC
pGL3-SPOP-Mut1-Luciferase	TCCCTCGACTCTCCTAAAAAAACTCACCTGAGAGCGGC	GCCGCTCTCAGGTGAGTTTTTTTAGGAGAGTCGAGGGA
pGL3-SPOP-Mut2-Luciferase	CTCTTCAGGGAGGTGAAAAAAAAAAACTCCGACAGGACAACC	GGTTGTCCTGTCGGAGTTTTTTTTTTTCACCTCCCTGAAGAG
pGL3-SPOP-Mut3-Luciferase	GTATATGTGTGTGGCTTTTTTTGGGGGGACAGTGGGGG	CCCCCACTGTCCCCCCAAAAAAAGCCACACACATATAC
pGL3-SPOP-Mut4-Luciferase	GATGGGGCTGGGAAATGATTTTTTTTCTGAAGGCAGGGGGC	GCCCCCTGCCTTCAGAAAAAAAATCATTTCCCAGCCCCATC
siRNAs		
CUL3-1	AACAACUUUCUUCAAACGCUA	UAGCGUUUGAAGAAAGUUG
CUL3-2	AACAACACUUGGCAAGGAGAC	GUCUCCUUGCCAAGUGUUG
RBX1-1	GGGAUAUUGUGGUUGAUAA	UUAUCAACCACAAUAUCCC
RBX1-2	GGAACCACAUUAUGGAUCU	AGAUCCAUAAUGUGGUUCC
SPOP	GCAAACGCCUGAAGCAAUC	GAUUGCUUCAGGCGUUUGC
VEZF1-1	GUGGGAAGAAGCUGUUAAA	UUUAACAGCUUCUUCCCAC
VEZF1-2	GUGCACCAGAAACAUUAAA	UUUAAUGUUUCUGGUGCAC

Supplementary Table S2. The sequences of primers and siRNAs (*Cont'd*)		
**Name**	**Forward primer (5'-3')**	**Reverse primer (5'-3')**
RT-qPCR		
ACTB	CACTCTTCCAGCCTTCCTTC	GTACAGGTCTTTGCGGATGT
CCL1	CTCATTTGCGGAGCAAGAGAT	GCCTCTGAACCCATCCAACTG
CCL11	CCCCTTCAGCGACTAGAGAG	TCTTGGGGTCGGCACAGAT
CCL13	CTCAACGTCCCATCTACTTGC	TCTTCAGGGTGTGAGCTTTCC
CCL15	TCCCAGGCCCAGTTCATAAAT	TGCTTTGTGAGATGTAGGAGGT
CCL19	TACATCGTGAGGAACTTCCACT	CTGGATGATGCGTTCTACCCA
CCL2	ATCACCAGCAGCAAGTGTCC	TGGGTTGTGGAGTGAGTGTT
CCL20	TGCTGTACCAAGAGTTTGCTC	CGCACACAGACAACTTTTTCTTT
CCL21	GTTGCCTCAAGTACAGCCAAA	AGAACAGGATAGCTGGGATGG
CCL22	ATCGCCTACAGACTGCACTC	GACGGTAACGGACGTAATCAC
CCL23	CATCTCCTACACCCCACGAAG	GGGTTGGCACAGAAACGTC
CCL24	ACATCATCCCTACGGGCTCT	CTTGGGGTCGCCACAGAAC
CCL25	GGCCCTCATGCTGTAAAGAAG	TGCTGATGGGATTGCTAAACTT
CCL27	GCAGCATTCCTACTGCCAC	AGGTGAAGCACGAAAGCCTG
CCL3	AGTTCTCTGCATCACTTGCTG	CGGCTTCGCTTGGTTAGGAA
CCL4	CTGTGCTGATCCCAGTGAATC	TCAGTTCAGTTCCAGGTCATACA
CCL5	ATATTCCTCGGACACCACACCC	CTCTGGGTTGGCACACACTT
CCL8	TGGAGAGCTACACAAGAATCACC	TGGTCCAGATGCTTCATGGAA
CD14	ACGCCAGAACCTTGTGAGC	GCATGGATCTCCACCTCTACTG
CSF-1	GTGAAAGTTTGCCTGGGTCC	ACAGACCAACAACAGCAGGG
CSF-2	CCTGAGTAGAGACACTGCTGC	TCTGGGTTGCACAGGAAGTT
CSF-3	GCTGCTTGAGCCAACTCCATA	GAACGCGGTACGACACCTC
CX3CL1	CGCGCAATCATCTTGGAGAC	CCACAGACTCGTCCATTCCC
CXCL1	ATTGTGAAGGCAGGGGAATGT	GCCCCTTTGTTCTAAGCCAGA
CXCL9	CCAGTAGTGAGAAAGGGTCGC	AGGGCTTGGGGCAAATTGTT
CXCL10	GTGGCATTCAAGGAGTACCTC	TGATGGCCTTCGATTCTGGATT
CXCL11	GACGCTGTCTTTGCATAGGC	GGATTTAGGCATCGTTGTCCTTT
CXCL12	ATTCTCAACACTCCAAACTGTGC	ACTTTAGCTTCGGGTCAATGC
CXCL13	GCTTGAGGTGTAGATGTGTCC	CCCACGGGGCAAGATTTGAA
CXCL16	CCCGCCATCGGTTCAGTTC	CCCCGAGTAAGCATGTCCAC
CXCL2	TGTGACGGCAGGGAAATGTA	TGCTCTAACACAGAGGGAAACA
CXCL5	TGTGCAATTAACAAAGCTACTGC	ACTCATAACAAAATGGTCAAAACCT
CXCL6	AGAGCTGCGTTGCACTTGTT	GCAGTTTACCAATCGTTTTGGGG
FAS	TCTGGTTCTTACGTCTGTTGC	CTGTGCAGTCCCTAGCTTTCC
HIF1A	CACCACAGGACAGTACAGGAT	CGTGCTGAATAATACCACTCACA
IL-1α	AGATGCCTGAGATACCCAAAACC	CCAAGCACACCCAGTAGTCT
IL-1β	ATGATGGCTTATTACAGTGGCAA	GTCGGAGATTCGTAGCTGGA
IL-11	CGAGCGGACCTACTGTCCTA	GCCCAGTCAAGTGTCAGGTG
IL-15	TTGGGAACCATAGATTTGTGCAG	GGGTGAACATCACTTTCCGTAT
IL-16	GCCGAAGACCCTTGGGTTAG	GCTGGCATTGGGCTGTAGA

Supplementary Table S2. The sequences of primers and siRNAs (*Cont'd*)

Plasmid	Forward primer (5'-3')	Reverse primer (5'-3')
RT-qPCR (Cont'd)		
IL-1ra	CATTGAGCCTCATGCTCTGTT	CGCTGTCTGAGCGGATGAA
IL-22	GCTTGACAAGTCCAACTTCCA	GCTCACTCATACTGACTCCGT
IL-24	TTGCCTGGGTTTTACCCTGC	AAGGCTTCCCACAGTTTCTGG
IL-32	TGGCGGCTTATTATGAGGAGC	CTCGGCACCGTAATCCATCTC
IL-33	GTGACGGTGTTGATGGTAAGAT	AGCTCCACAGAGTGTTCCTTG
IL-6	AGACAGCCACTCACCTCTTCAG	TTCTGCCAGTGCCTCTTTGCTG
IL-6r	CATGTGCGTCGCCAGTAGT	AGCTCAAACCGTAGTCTGTAGA
IL-7	TTGGACTTCCTCCCCTGATCC	TCGATGCTGACCATTAGAACAC
IL-8	ACACTGCGCCAACACAGAAA	TTGCTTGAAGTTTCACTGGCATC
iNOS	TTCAGTATCACAACCTCAGCAAG	TGGACCTGCAAGTTAAAATCCC
PARP14	TGTTAGTGGAGAACATAAGTGGC	TGAATGGTGCTTGGTACAATCAT
PDGFβ	CTCGATCCGCTCCTTTGATGA	CGTTGGTGCGGTCTATGAG
SERPINB9	AATGCAAGTGGTACTTTTGCCA	AAGCCCGATGAATGTCTTCCT
SOX2	GCCGAGTGGAAACTTTTGTCG	GGCAGCGTGTACTTATCCTTCT
SPOP	GCCCTCTGCAGTAACCTGTC	GTCTCCAAGACATCCGAAGC
TGFβ2	CAGCACACTCGATATGGACCA	CCTCGGGCTCAGGATAGTCT
TNF	CCTCTCTCTAATCAGCCCTCTG	GAGGACCTGGGAGTAGATGAG
VEGFA	AGGGCAGAATCATCACGAAGT	AGGGTCTCGATTGGATGGCA
VEZF1	TTGGGAAAACCCAGTTCCCC	GCTTCATGGGCTGCCATTTT
ChIP		
VEZF-SPOP 1	AGGCGACAGCTCAGTATTTG	CCGTCCCTTCTCCCTATCTCA
VEZF-SPOP 2	CATGGCGTCAGCACGTC	GCGCACTAGGAAAGATAGGCG
VEZF-SPOP 3	GGGTGTAGGAAATGGGACTGAG	CAGTATGAGGCAAGCGCAG

Supplementary Table S3. List of antibodies, chemicals and kits

Reagents	Company	Cat #	Dilution	RRID
Antibodies				
anti-CD68	Cell Signaling Technology	76437S	IF (1:200)<br>IHC (1:1,000)	AB_2799882
anti-CK5	COVANCE	PRB-160P	IF (1:1,000)<br>IHC (1:2,000)	AB_10063444
anti-CUL3	Abways	CY7196	WB (1:1,000)	NA
Anti-F4/80	Bioworld Technology, Inc.	RB0001	IF (1:50)	NA
anti-FLAG	Sigma-Aldrich	F3040	WB (1:5,000)<br>IF (1:500)<br>IP (1:1,000)	AB_439712
anti-GAPDH (6C5)	Santa Cruz	sc-32233	WB (1:1,000)	AB_627679
anti-HA (F-7)	Santa Cruz	sc-7392	WB (1:1,000)<br>IP (1:200)	AB_627809
anti-Integrin β1	Cell signaling	34971S	WB (1:1,000)	AB_2799067
anti-IL-6 neutralizing antibody	R&D systems	MAB2061	Cell assay<br>(100 ng/ml)	AB_2127616
anti-KLF4	Proteintech	11880-1-AP	WB (1:1,000)	AB_10640807
anti-Myc (9E10)	Santa Cruz	sc-40	WB (1:1,000)	AB_627268
anti-RBX1	HuaBio	ER1802-9	WB (1:1,000)	AB_3069189
anti-SPOP	Proteintech	16750-1-AP	WB (1:1,000)	AB_2756394
anti-SPOP	Abcam	ab168619	IHC (1:100)<br>IF (1:50)	NA
anti-STAT3	Cell Signaling Technology	9139S	WB (1:1,000)<br>IP (1:200)	AB_331757
anti-p-STAT3(Y705)	Cell Signaling Technology	9145S	WB (1:1,000)	AB_2491009
anti-VEZF1 (B-4)	Santa Cruz Biotechnology	sc-365560	WB (1:1,000)<br>IHC (1:1,000)<br>ChIP (2 μg)	AB_10846717
ABflo® 488-conjugated Goat anti-Rabbit IgG (H+L)	ABclonal	AS053	IF (1:1,000)	AB_2768320
ABflo® 555-conjugated Goat anti-Mouse IgG (H+L)	ABclonal	AS057	IF (1:1,000)	AB_2768322
Alexa Fluor® 647-conjugated Goat anti-Rat IgG	Abcam	ab150167	IF (1:1,000)	AB_2864291
Protein A/G PLUS-Agarose	Santa Cruz Biotechnology	sc-2003	CoIP (20 μl/test)	AB_10201400
Chemicals and Kits				
Blasticidin S	Yeasen	60218ES60	20 μg/ml	
ClonExpress MultiS One Step Cloning kit	Vazyme	C113-02	NA	
Cycloheximide (CHX)	Sigma-Aldrich	66-81-9	100 μg/ml	
DAB kit	MXB Biotechnologies	DAB-2031	NA	
DAPI	BBI Life Science	28718-90-3	5 μg/ml	
DH5α competent cells	Sangon Biotech	B528413-0020	NA	
DMSO	Sigma-Aldrich	D2650	NA	
EGF, human recombinant protein	PeproTech	AF-100-15	10 ng/ml	
EZ-ChIP kit	Millipore	17-295	NA	
bFGF, human recombinant protein	PeproTech	100-18B	10 ng/ml	
Human CCL2/MCP-1 ELISA kit	MultiSciences (Lianke) Biotech	EK1872	NA	
Human IL-6 ELISA kit	ExCell Bio	EH004-96	NA	
IL-6 protein, human recombinant protein	PeproTech	200-6	50 ng/ml	
Lipofectamine 2000	ThermoFisher Scientific	11668019	NA	
Luciferase assay system	Promega	E1501	NA	

Supplementary Table S3. List of antibodies, chemicals and kits (*Cont'd*)

Reagents	Company	Cat #	Assay (Dilution)	RRID
Chemicals and Kits				
Matrigel	Corning	354234	NA	
MG132	Selleck	S2619	10 μM	
MTT	Sigma-Aldrich	M2128	0.5 mg/ml	
Mut Express II Fast Mutagenesis kit V2	Vazyme	C214-01	NA	
NP-40 lysis buffer	Beyotime	P0013F	NA	
Prime-Script RT-PCR kit	Takara	RR047	NA	
PrimeSTAR MAX DNA polymerase	Takara	R045A	NA	
Puromycin dihydrochloride	Yeasen	60210ES80	2 μg/ml	
PVDF membrane	Millipore	IPVH00010	NA	
RS 504393	MedChemExpress	HY-15418	2 μM	
Stattic	MedChemExpress	HY-13818	20 μM	
Super Signal West Pico Stable Peroxide Solution	ThermoFisher Scientific	180-5001E	NA	
SYBR qPCR Master Mix (High Rox)	Vazyme	Q341-02	NA	
TRIzol	Life Technologies	15596018	NA	
T4 ligase	ThermoFisher Scientific	EL0011	NA	

Notes: NA, not applicable.
